# Medicinal Plants as Biopesticides Against Pests and Diseases of Maize (*Zea mays* L.) in Africa: Ethnobotanical Insights and Challenges

**DOI:** 10.3390/plants15101549

**Published:** 2026-05-19

**Authors:** Florence Bukky Aina, Lisa Buwa-Komoreng, Lelethu Unathi-Nkosi Peter Heshula, Charles Shelton Mutengwa

**Affiliations:** 1 Department of Agronomy, University of Fort Hare, Private Bag X1314, Alice 5700, South Africa; cmutengwa@ufh.ac.za; 2 SAMRC Microbial Water Quality Monitoring Centre, University of Fort Hare, Private Bag X1314, Alice 5700, South Africa; lheshula@ufh.ac.za; 3 Department of Botany, University of Fort Hare, Private Bag X1314, Alice 5700, South Africa; lbuwa@ufh.ac.za; 4 Department of Zoology & Entomology, University of Fort Hare, Private Bag X1314, Alice 5700, South Africa

**Keywords:** biopesticides, medicinal plants, maize, phytochemicals, ethnobotany, pests, diseases

## Abstract

Maize (*Zea mays* L.) is a significant staple food crop in the developing world. Despite its significance, diseases and pests are limiting its supply. Farmers have primarily relied on synthetic chemicals as control measures; however, these chemicals are harmful to humans, animals, and the environment and exacerbate pest recurrence. Medicinal plants have shown promising potential as alternative pest- and disease-controlling agents, offering an economical, sustainable, biodegradable, and cost-effective approach. This review article synthesises phytochemical, ethnobotanical, and experimental data from relevant peer-reviewed papers published across various years to identify medicinal plants. Thirty-one unique plant families have been identified and have been used to control pests and diseases of maize. Some families represented both antifungal and insecticidal applications. Medicinal plants such as *Senna obtusifolia*, *Euphorbia balsamifera*, *Aristolochia ringens*, *Allium sativum*, *Azadirachta indica*, *Carica papaya*, *Moringa oleifera*, and *Ficus exasperata* have shown antifungal and insecticidal properties, primarily under laboratory conditions. Most of the evidence is derived from laboratory studies, with only limited validation in real field conditions and with limited evaluation of safety for non-target organisms. Furthermore, this review highlighted the extraction methods, solvents used, plant parts, major active ingredients, and mode of action. Future prospects for integrating ethnobotanical knowledge with contemporary scientific methods to optimise biopesticide production are also discussed, along with the challenges of standardisation, formulation, and commercialisation.

## 1. Introduction

Maize (*Zea mays*) belongs to the Poaceae family, specifically to the *Zea* genus within the Andropogoneae tribe and the Panicoideae subfamily [1]. It is a major food source for human consumption in many nations, particularly in Sub-Saharan Africa (SSA), Latin America, and a few Asian countries, accounting for more than 20% of total calorie intake [2]. Maize is a crop that is more adaptable and multipurpose than rice and wheat [2,3]. Although it is widely used in industrial processing and the production of bioenergy, the most common use in industrialised economies is for animal feed [4]. Asia provides a prime example of how the demand for animal-source foods has increased due to economic development, characterised by income growth and urbanisation [5]. As a result, more maize is being used as feed. Consequently, maize plays a dynamic and varied function in global agri-food systems, supporting both food and nutrition security [2]. Animal feed currently uses over 800 million tonnes of cereals, or one-third of all cereal production, and is expected to reach over 1.1 billion tonnes by 2050. The demand for maize and coarse grains is expected to account for more than half of grain production in 2050, driven by the growth of the monogastric sector [6]. It is anticipated that maize acreage will surpass wheat acreage by 2030, reaching 227 million acres [7]. However, it is estimated that the world’s cereal production, primarily from rice and maize, will rise by about 320 million tons to 3.1 billion tons by 2032 [8].

For a long time, pests, including weeds, insects, and viruses, have been major dangers to agriculture, significantly lowering agricultural production. Bacteria, fungi, weeds, and insects are among the biggest threats to food security and sustainable farming since they cause over 40% of agricultural productivity losses worldwide [9]. Farmers have historically used synthetic insecticides to manage diseases and pests in various areas. However, overuse of these pesticides has resulted in several issues, such as insect resistance, environmental contamination, and risks to human health [10].

*Sitophilus zeamais*, one of the most destructive insects, primarily causes losses in tropical areas due to favourable growth conditions [11]. The damage begins when adults puncture the grain to lay eggs, while the larval larvae create holes in the endosperm to feed. Storage losses can vary from 20 to 60% if not properly managed, or more in extreme situations [11]. Fungal diseases and insect pests pose serious risks to maize production in SSA due to their destructive nature and capacity to result in large yield losses, particularly in favourable weather conditions [12].

Some plants have been used for protection against insects for over 3000 years [13]. Smallholder farmers in Southeast Asia, Latin America, and Africa have embraced indigenous knowledge and methods of controlling pests and diseases with plants and their extracts [14]. Biopesticides are ecologically friendly and host-specific; hence, they are far more beneficial to utilise than traditional chemical alternatives [15]. Biopesticides are very promising because they not only control the pests but also decrease the adverse effects of synthetic chemical pesticides [16]. As a subclass of biopesticides, nano-biopesticides are a cutting-edge class that combines biological agents with nanomaterials for improved performance. These creative formulations utilise the power of nanomaterials to develop biopesticide delivery systems that are both efficient and targeted [16].

The most significant obstacle to the commercialisation of novel botanical pesticides is still regulatory approval. This is still the case today. The burden of registering new botanical insecticides has grown in the United States and the European Union, where it now takes about two and four years, respectively, from data submission to approval. However, some jurisdictions, such as China and Korea, have regulatory schemes that are more favourable to the approval of plant extracts or oils [17].

The purpose of this review is to compile, analyse, and synthesise experimental and ethnobotanical data on a few therapeutic plants that have historically been utilised in Africa to manage diseases and pests associated with maize. In addition to highlighting their bioactive substances, mechanisms of action, and useful applications, it also suggests ways of incorporating these plant-based products into environmentally friendly pest control methods and identifies obstacles to doing so. The focus is on indigenous and therapeutic plants that are used to control maize diseases and pests in the field and during storage. This includes both conventional and scientifically proven uses of insecticides derived from plants.

This review was conducted as a narrative review of existing knowledge on medicinal plants used to manage maize pests and diseases. A wide search was conducted across major academic databases such as Google Scholar, Scopus, and Web of Science. The search strategy combined keywords like “medicinal plants,” “biopesticides,” “maize pests,” “maize diseases,” “ethnobotany,” “plant extracts,” and “*Sitophilus zeamais*” to capture the most relevant studies. Studies were selected based on their scientific merit, topic relevance, and contribution to experimental validation or ethnobotanical knowledge. Peer-reviewed articles were given priority, and when needed, both current research and foundational earlier works were included. As a narrative review, this study does not follow a formal systematic review protocol or apply strict inclusion/exclusion criteria or quantitative bias assessments. Instead, the study aimed for broad coverage of the literature and to reduce selection bias by consulting multiple sources.

## 2. Impact of Pests and Diseases on Maize Yield and Storage Losses

Global agriculture is at risk of plant diseases and alien insect pests, which account for 40% of crop production losses annually, or approximately $220 billion. Insects that damage crops alone cause economic losses of $70 billion [18]. Crop production is constrained by various organisms, including competitive plants, vertebrate pests (such as birds, mammals, and rodents), diseases (viruses, phytoplasma bacteria, and fungi), and invertebrates (molluscs, mites, nematodes, and insects). These harmful organisms can grow on seeds or other stored food products, as well as on plants in the field and greenhouses [19]. Globally, storage pests severely damage dried and stored food items, including grains, pulses, processed foods, as well as non-food products derived from these items [20]. Postharvest losses from storage insects might reach 20% in underdeveloped countries and 9% in developed ones. Additionally, storage pests introduce dead and live insects, body fragments, and chemical waste to the products, thus contaminating them [20].

The production of maize is threatened by several factors, the most significant of which are biotic factors. It has been demonstrated that biotic elements, particularly weeds such as *Striga* and insects including stem borers, can significantly impact maize yield, causing up to 30% losses [21,22]. Significant reductions in maize productivity in the affected areas have also been linked to pathogens such as wheat streak mosaic virus, sugarcane mosaic virus, and maize dwarf mosaic virus [22]. Biotic stressors, such as diseases and pests, have a significant impact on global agricultural production, resulting in annual losses of up to $220 billion [21]. Pests have the potential to severely damage products stored in warehouses. Pest-related storage losses threaten the livelihoods of farmers throughout Africa [21]. A study conducted in Tanzania reported that the main obstacles to maize production that farmers identified were insect pests in the field (90.1%), *Striga* infestation (93.1%), storage pests (72.7%), and drought (97.2%) [23].

Lepidopteran stem borers are one of the insect pests that severely reduce maize yields. The two most commercially significant maize stem borers in Sub-Saharan Africa are the African stemborer, *Busseola fusca* (Fuller), and the spotted stemborer, *Chilo partellus* (Swinhoe) [21,24,25]. When estimating yield losses based on farmers’ reports, it is likely to overestimate damage, especially for a pest like *Spodoptera frugiperda*, whose primary damage is defoliating maize with relatively less damage to other parts of the plant, in contrast to maize stem and ear borers, particularly the African stalk borer, which can cause equal damage to leaves, stems, and ears [24]. The fall armyworm (FAW), *Spodoptera frugiperda*, is a polyphagous pest that consumes over 60 different plant species; however, it has primarily affected certain crops, including maize, rice, millet, and sorghum [22,26]. All plant parts and various phases of maize growth are affected by FAW damage. The pest has been known to damage the stem base of seedlings, the growing stage of the leaves, and mature maize plants, where it can feed on tassels or penetrate the ears [27,28]. It has been demonstrated that damage to the kernel increases the growth of several fungi that produce mycotoxins [22]. Dark brown or grey necrotic and chlorotic lesions, or discoloured areas surrounding the infection site, are common symptoms of fungal pathogen-induced diseases in maize leaves [29]. Furthermore, malformations, stunting or dwarfing, discolouration, and necrosis are the main symptoms of viral infections in maize. Most viral infections are transmitted to plants by insects that feed on the leaves [29].

One of the main impediments to food security in poor nations is post-harvest losses. Hence, grain loss stemming from poor post-harvest handling procedures is one of the key barriers to ensuring food security in Africa [30,31,32]. Research indicates that inadequate storage facilities and poor post-harvest handling practices have significantly contributed to the contamination of maize grain with mycotoxins from fungal diseases [33,34]. Insect pest infestations are made worse by extreme temperatures and unfavourable warehousing conditions [34]. Traditional preservation techniques are seriously threatened by mycotoxin contamination, particularly with regard to maize, which renders the food unfit for human or animal consumption. Mycotoxins damage the food chain and reduce the quality of seeds [31].

Sola et al. stated that the most common pests that cause the greatest damage and post-harvest losses are bruchids (*Callosobruchus* spp. and *Acanthoscelides obtectus*), weevils (*Sitophilus* spp.), larger grain borer (*Prostephanus truncatus*), lesser grain borer (*Rhyzopertha dominica*), and flour beetles (*Tribolium* spp.) [35,36,37]. The primary harmful pests of maize storage products include *Sitophilus oryzae* (L.), *Tribolium castaneum* (Herbst), *Oryzaephilus surinamensis*, *Trichoderma* granarium, *Cadra contella*, *Sitophilus zeamais* (Motsch.), *Cryptolestes pusillus*, *Sitotroga cerealella* (Oliver), *Tribolium confusum*, *Rhizopertha dominica* (Fabricius), *Sitophilus granarius*, *Cryptolestes ferrugineus*, *Plodia interpunctella*, and *Corcyra cephalonica* (Stainton) [38,39,40].

The maize weevil (*Sitophilus zeamais)* is a primary storage pest of maize in the tropics. It is considered a cosmopolitan storage pest and is widely dispersed in tropical countries [41,42]. Significant grain damage can have an adverse effect on human health, in addition to reducing the nutritional value, weight, and germination rates of seeds. Additionally, harmful fungi, such as *Aspergillus flavus*, which is associated with several bacterial species, can be spread by insects [43]. The majority of grain damage is caused by maize weevil larvae and adults [43,44]. Fifty per cent of maize weevil eggs may be laid within the first five weeks of an adult’s life. Grain is drilled by the female to form small, chewed egg-laying chambers, which are subsequently closed by a secretion to protect the organism and allow it to complete its developmental process [43].

## 3. Limitations of Synthetic Chemicals

In agriculture, synthetic chemicals are used to control weeds, pests, and plant diseases, thereby improving plant protection and increasing crop yields. There are many different types of pesticides, including nematicides and rodenticides [45,46]. Furthermore, they are believed to enhance public health by increasing food production and reducing food-borne and vector-borne diseases caused by bacteria, fungi, or other pathogens [47]. However, pesticides affect the heart, liver, kidneys, and reproduction, causing infertility [46]. Adverse environmental impacts are associated with active ingredients such as mirex, dichlorodiphenyltrichloroethane (DDT), endrin, chlordane, hexachlorobenzene, and dieldrin [45]. A major concern for humans and other terrestrial ecosystems is the persistent pollution of the environment by pesticides, which enter the food chain and accumulate in the soil [45].

Typically, pesticides are poisonous substances that can have significant adverse consequences even in minute quantities. Every year, almost 3 million people become poisoned by pesticides, which results in about 200,000 fatalities globally [48]. Pesticides used as plant protectants on agricultural fields can enter nearby water bodies by groundwater inflow, soil erosion, surface runoff, spray drift, and surface drainage systems [49]. The skin, eyes, mouth, and nose are all possible entry points for the chemicals [50]. Improper use of pesticides is hazardous to humans, plants, animals, and the environment [51,52]. Organochlorinated pesticides, heavy metals, personal hygiene items and pharmaceuticals, phosphates and nitrates have all been found in varying amounts in the surface waters of the uMsunduzi River in South Africa [53].

Insecticide-based chemical treatment is often regarded as an efficient method of managing pests. However, due to the development of resistance to the active chemicals, the use of insecticides may not always be effective [19,54]. Smallholder farmers lack access to adequate information on pesticide handling, and they are also at risk of hazardous exposure due to a lack of access to protective gear [55]. Moreover, environmental pollution from these chemicals poses a danger to people and livestock through contaminated food and agricultural residues. Additionally, chemical pesticides have unintended negative effects on beneficial non-target species, and pesticide resistance is commonly observed [56,57].

Pesticide exposure may increase the incidence of type 2 diabetes mellitus, especially exposure to organochlorine insecticides and some organophosphate compounds [58,59]. Some pesticides are long-lasting pollutants in the environment because they refuse to decompose [60]. The rate at which a pesticide degrades depends on the microbial biomass, pH of the soil, organic matter content and other environmental factors such as temperature, climatic conditions, and moisture [61].

## 4. The Role of Plant-Based Insecticide

The use of chemical pesticides for managing insect pests has become a major global concern due to environmental contamination, limited specificity in mode of action, the emergence of insect resistance, and residual effects [62]. Pest control is crucial to the health and productivity of humans, animals, and plants. Novel pest control agents may be discovered by evaluating plant extracts with proven therapeutic properties for pesticidal efficacy. A global search for alternatives to chemical pesticides is being undertaken through the assessment of the effectiveness of natural products for crop protection and pest control. Botanicals and other biopesticides offer a viable alternative to synthetic pesticides. This is because they are easily accessible, reasonably priced, safe, biodegradable, and environmentally friendly [18,63,64].

Biopesticides are natural pesticides derived from materials produced by living organisms, including bacteria, plants, animals, and genetically modified organisms [65,66]. Three primary categories of biopesticides exist: microbial pesticides, which involve bacteria, algae, fungi, viruses, or protozoa as active ingredients; biochemical pesticides, which are naturally produced compounds that use non-toxic methods to control pests; and plant-incorporated protectants (PIPs), which are compounds made by plants as a result of genetic modifications [67,68,69]. The number of plant species classified as botanical pesticides is approximately 1079, and 866 of these species exhibit physiological, repellent, toxicological, and deterring effects on insects [70].

Before the development of pharmaceutical drugs and iatrochemistry in the sixteenth century, people used plants to treat and prevent diseases in both humans and animals. [71]. However, it has been demonstrated that many plants produce secondary metabolites that protect humans, animals, and plants against parasites, bacteria, fungi, and insects [62]. The Food and Drug Administration (FDA) of the United States stated that essential oils, or botanical pesticides, are safer than conventional pesticides, which are said to have teratogenic, neurotoxic, carcinogenic, and mutagenic effects on non-target species in addition to raising the risk of ozone depletion [72,73]. Additional issues include the government’s estimated loss of several billion dollars globally in environmental and societal damages resulting from the negative effects of pesticides on public health, as well as losses of livestock and crops and their products, pollination issues, honeybee losses, the devastation of natural enemies, and the loss of fish and other wildlife [73].

Many plant parts, including bark, leaves, stems, fruits, cloves, rhizomes, flowers, grains, and seeds, contain phytochemical compounds that are allelochemicals, including alkaloids, amines, non-protein amino acids, cyanogenic glycosides, glucosinolates, lectins, and proteases [73,74]. These compounds have been shown to have insecticidal effects, including preventing feeding, regulating growth, repelling insects, suppressing oviposition (egg deposition), acting as a sterilant or toxin, and preventing the growth or death of insect pests [73]. For instance, organic farmers and eco-conscious consumers are particularly interested in using essential oils derived from aromatic plants. On a range of insects, they have growth-reducing, oviposition-inhibiting, growth-inhibiting, ovicides, insecticidal, antifeedant, and repelling properties [75].

## 5. Limitations and Challenges of Plant-Based Biopesticides

The effectiveness of biopesticides depends on the method of application and formulation, as well as the disease or pest type [66]. Because biopesticides are generated from living organisms (bacteria, fungi, viruses, protozoa) or natural metabolites, they often have shorter shelf lives than synthetic pesticides. Due to their intrinsic sensitivity to environmental elements, including heat, light, and moisture, these components deteriorate more quickly. Because of this, biopesticides usually require cool, dry, and dark conditions to be effective; any deviation reduces their field efficacy [66]. The instability of the ingredients in essential oil-based biopesticides may cause airborne drift. Evaporating essential oils has been shown to raise concentrations of carbon dioxide, carbon monoxide, and volatile organic compounds (VOCs) in indoor settings. It may also encourage the production of secondary pollutants like formaldehyde and secondary organic aerosols (SOAs) through reactions with oxidants and ozone [69]. Many people continue to doubt the effectiveness of biopesticides in comparison to chemical alternatives, frequently supposing them to be slower-acting. Price sensitivity is particularly important because biopesticides are frequently linked to more expensive organic products, which may cause a disconnect between a consumer’s ecological values and their ultimate purchase decision [76].

Large-scale commercial usage of biopesticides faces a number of challenges. These include their short shelf life; their inconsistent field efficacy, which is dependent on many abiotic conditions; and regulatory issues that prevent farmers from using them to control pests and diseases [77]. The development and commercialisation of biopesticides also necessitate large research and development expenditures as well as close observation to ascertain their efficacy in the field [77]. Effective risk communication is a crucial policy concern that is required to enable the safe and sustainable integration of biopesticides into agricultural systems, as their use has not yet taken centre stage in agricultural practice [69].

Although plant-based biopesticides have ecological benefits, they have several severe issues that restrict their use in agricultural systems. Among the primary constraints, one must include the variability of efficacy as a result of variation in the phytochemical composition because of the different plant species, geographical origin, harvesting conditions and extraction methods. This variability reduces the reproducibility, making it difficult to achieve product standardisation. The presence of environmental instability also restricts the efficacy of plant-based compounds further. Most bioactive constituents are very sensitive to light, temperature, and oxygen, thus degrading easily and decreasing their persistence under field conditions. This causes a high repetition rate that might require frequent application, which contributes to the labour and operation costs. The other significant hindrance is scalability. The transition to large-scale commercial production requires a large supply of raw materials, standardised processing, and quality requirements that are generally lacking in the current systems. Moreover, higher formulations and, more so, nanotechnology-based formulations are highly costly and may be unaffordable to the smallholder farmers. The other major issue is the dissimilarity between laboratory and field performance. Most of the plant extracts are very pesticidal in the controlled environment; however, their effectiveness in the field is normally less due to the complexity of their interaction with the environment and constraints in their use. In addition, the biopesticide regulations are either poorly developed or not cross-border-homogeneous, which further hinders commercialisation. To handle these issues, a multidisciplinary approach is needed that incorporates phytochemistry, formulation science, agronomy, and regulatory policy to enable the production of consistent and scalable plant-based pest management approaches.

## 6. Ethnobotanical-to-Biopesticide Development Workflow

**Figure 1:**
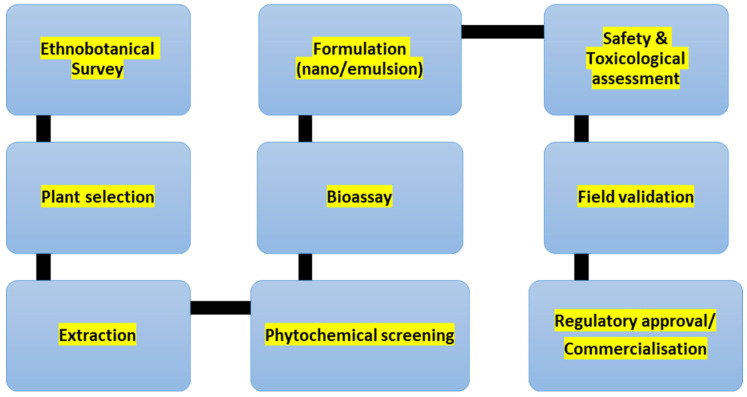
Ethnobotanical-to-biopesticide workflow chart.

A stage-gated pipeline combining phytochemical standardisation, mode-of-action bioassays, formulation science, and thorough safety evaluation is needed from ethnobotanical findings to deployable biopesticides (Figure 1). Although the efficacy in the laboratory is widely reported, the main bottlenecks in the African settings are the stability of the formulations, validation in the field, and the fragmented regulatory pathways. By using a coordinated roadmap, with a primary focus on reproducibility, quality control, and multi-location trials, it is possible to drastically translate plant-based pest control strategies into scalable and farmer-ready products.

## 7. Ethnobotanical Knowledge and Use of Medicinal Plants in Maize Protection

Ethnobotany is a branch of science that focuses on the relationship between humans and plants. In general, it encompasses the study, observation, and recognition of botanical biodiversity that is utilised to safeguard against and treat diseases in humans and cattle [78]. Since humans have always interacted with plants and had both direct and indirect effects on one another, such interaction is inevitable [79]. Understanding the interaction between plants and humans in particular habitats is largely dependent on ethnobotanical research [80]. Ethnobotany plays a crucial part in sustainable pest management by documenting and promoting the traditional knowledge of plants used by indigenous communities to control agricultural pests and diseases. Table 1 and Table 2 present medicinal plants traditionally utilised to manage maize diseases and pests in Africa, highlighting their ethnobotanical significance and potential as biopesticides. Several plants across 31 plant families have been used to control the maize weevil and pathogens of maize. Plants have been proven to be a good alternative as they are eco-friendly and readily available. Insecticidal efficacy of the reported medicinal plants in Table 1 was demonstrated mainly in the laboratory, and antifungal efficacy of the medicinal plants reported in Table 2 was demonstrated mainly as an in vivo, in vitro system, with a few demonstrations in the field. However, there is a gap between laboratory and field (practical) application; further studies should include field-based experiments to ascertain the effectiveness of the medicinal plants.

## 8. Phytochemical Constituents of Insecticidal and Antifungal Plants

Phytochemicals are naturally occurring substances that give plants their colour, flavour, and resistance to disease [98]. Currently, over 10,000 different types of phytochemicals have been identified [99]. Depending on their distinct chemical structures and biological roles, phytochemicals can be generally categorised into several types, including polyphenols, flavonoids, carotenoids, alkaloids, and glucosinolates [100,101]. They are present in food sources, including fruits, vegetables, whole grains, nuts, and herbs. These phytochemicals possess potent antioxidant properties, as well as antiviral, antimutagenic, antihelmintic, anticancer, antiallergic, antidiarrheal, anticarcinogenic, and antimicrobial properties [98,102,103].

**Figure 2:**
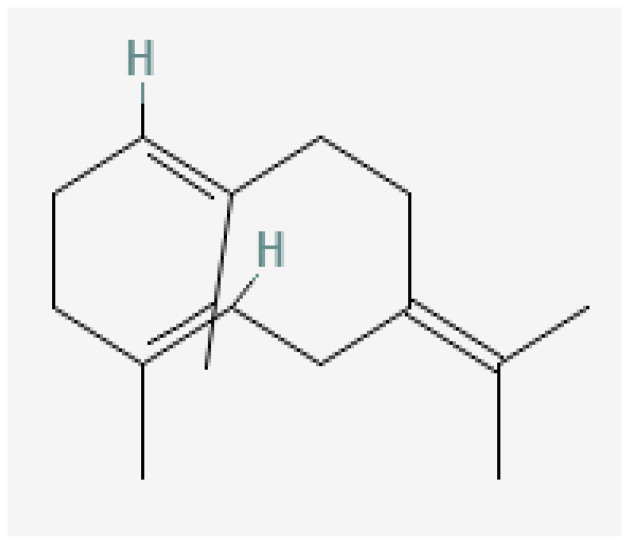
Germacrene-B.

**Figure 3:**
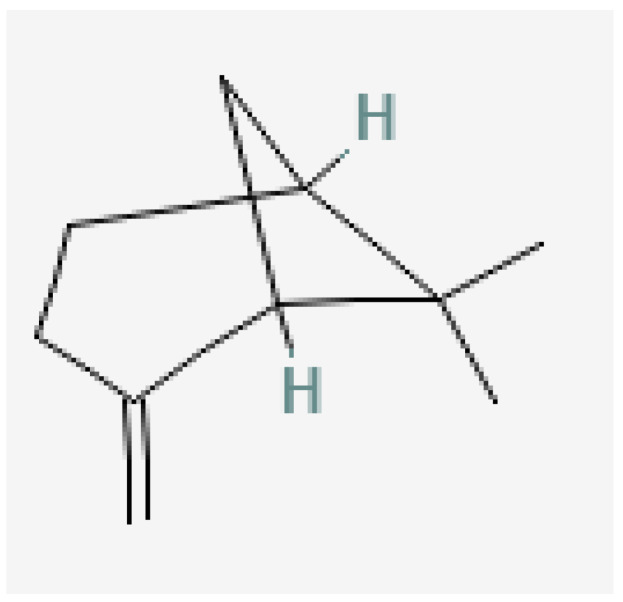
Beta-pinene.

**Figure 4:**
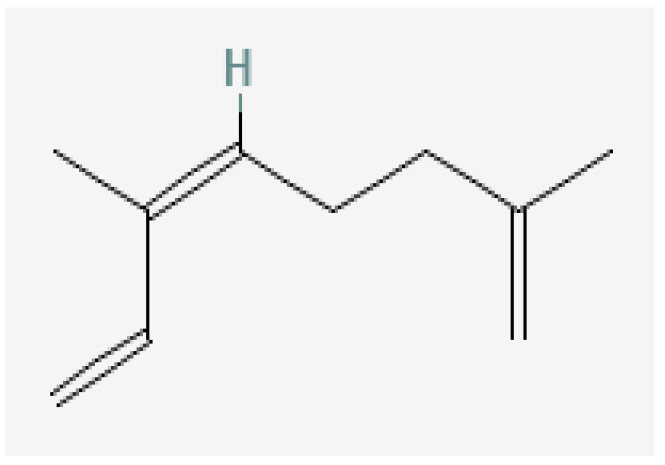
Ocimene.

**Figure 5:**
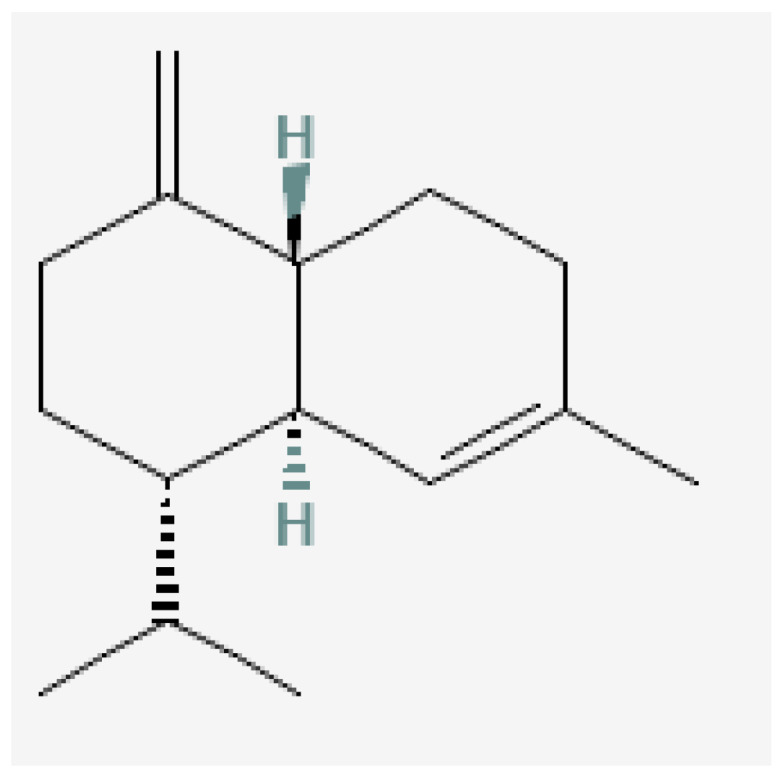
Gamma-cadinene.

**Figure 6:**
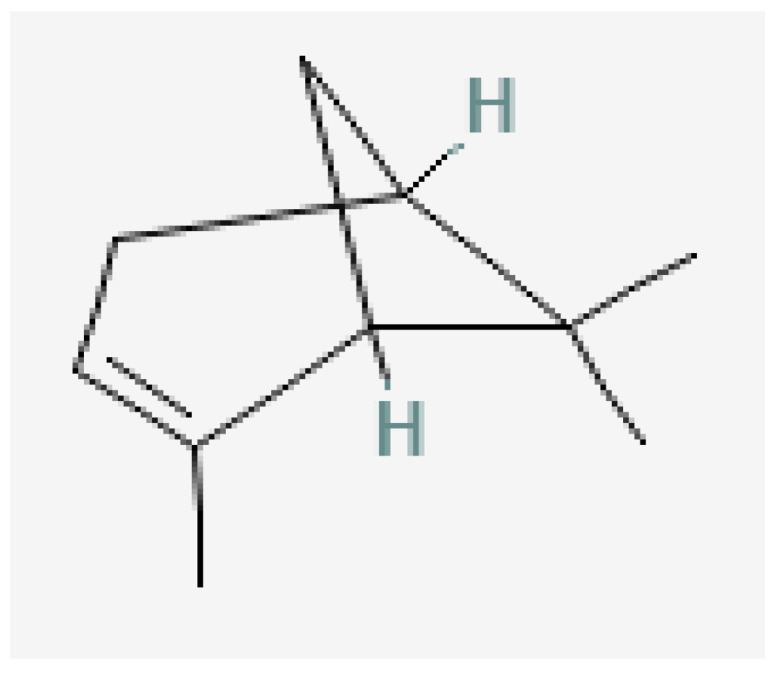
Alpha-pinene.

**Figure 7:**
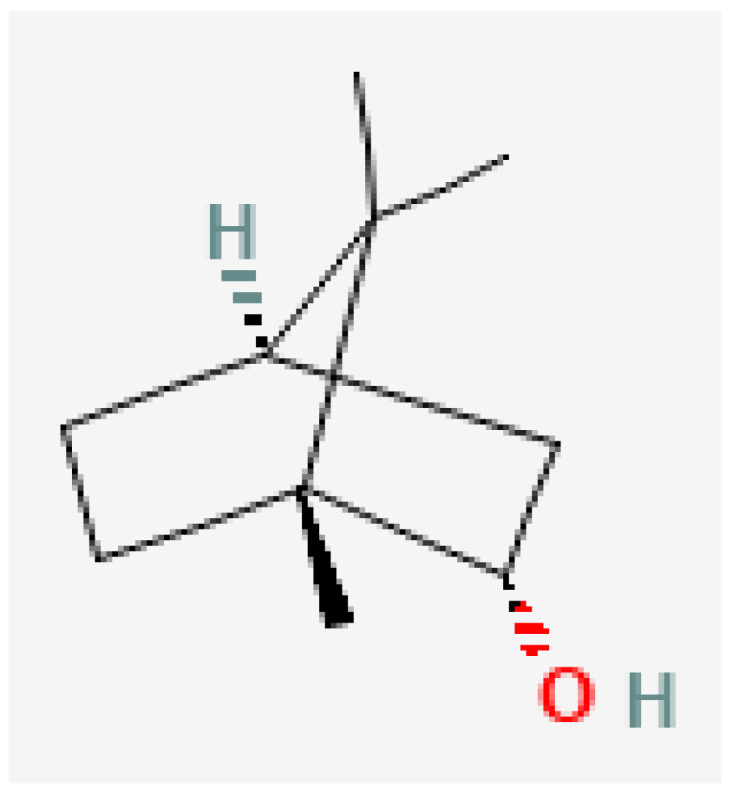
Borneol.

**Figure 8:**
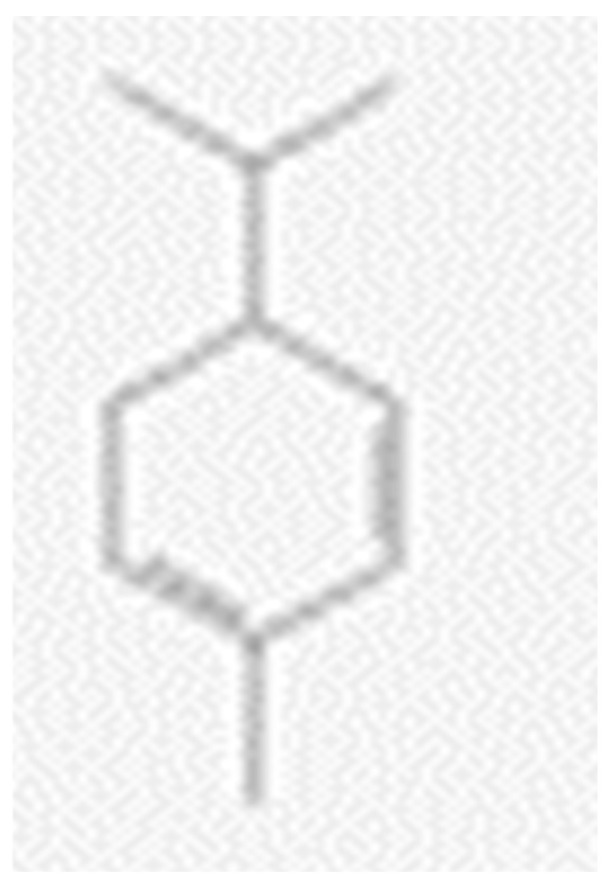
Alpha-phellandrene.

**Figure 9:**
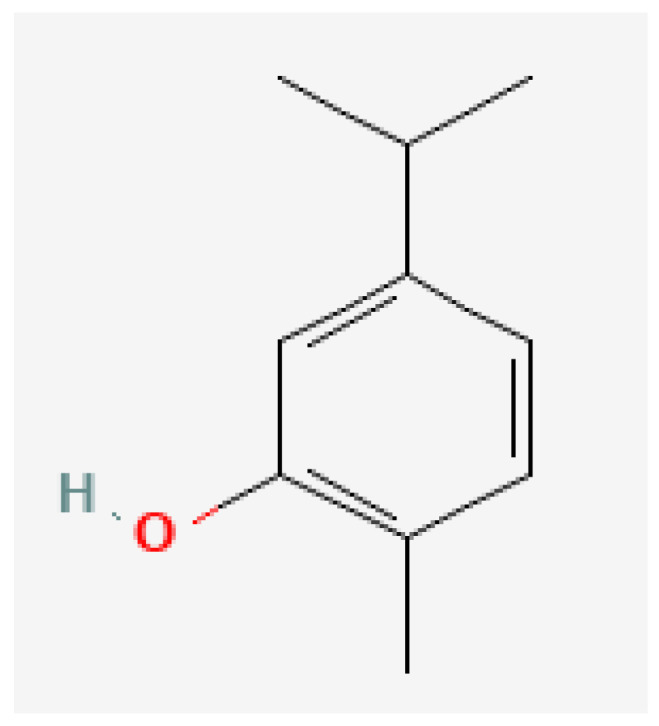
Carvacrol.

**Figure 10:**
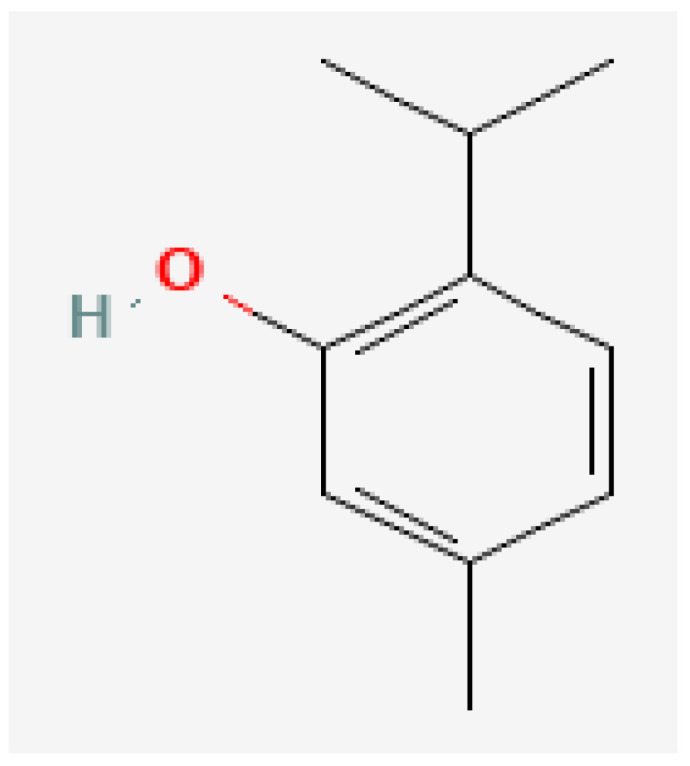
Thymol. Source: https://pubchem.ncbi.nlm.nih.gov/ (assessed on 14 April 2026).

Plant-based bioactive chemicals can be used in routine processes to reduce the requirement for conventional chemical treatments. It is possible to regulate the release of physiologically active components under field conditions, guarantee their stability, and improve their efficacy by using formulations that incorporate emulsifying agents, defoamers, surfactants, solvents, polymers, stabilisers, and other compounds [104]. Many secondary metabolites or bioactive compounds, including phenolic compounds, flavonoids, alkaloids, and essential oils (such as alpha-pinene, borneol, beta-pinene, alpha-phellandrene, germacrene B, gamma-cadinine, and ocimene (Figure 2, Figure 3, Figure 4, Figure 5, Figure 6, Figure 7 and Figure 8), which serve as the basis for insecticides, can be produced by medicinal plants [105]. Thymol and carvacrol (Figure 9 and Figure 10), two phenols found in EOs, can act as insecticides or repellents to control insects in grain storage [106].

The active ingredients in botanical pesticides, particularly the distinct structural patterns of secondary metabolites, such as alkaloids, essential oils, flavonoids, phenols, phytosterols, polyketides, and resins, are capable of providing antifungal, herbicidal, antibacterial, and insecticidal effects [107]. Phenolic compounds, including phenolic acid, coumarins, flavonoids, phenols, and tannins, are abundant in aromatic plants and are produced through the shikimate pathway and phenylpropanoid metabolism [104,108,109]. Fungi, bacteria, insects, nematodes, and weeds are all poisoned by these substances. Natural phenolics exhibit varying degrees of cytotoxicity, often in conjunction with apparently opposite properties, such as antioxidant activity. Phenolics work by altering the permeability of cell membranes and altering intracellular functions [104].

Plant parts, including seeds, leaves, roots, fruits, flowers, and stems, all contain phytochemicals. Nonetheless, the outer layers of plant tissues contain significant amounts of several phytochemicals, particularly colour pigments [110]. The potential of essential oils and plant extracts from various botanicals to protect crops from pests and diseases has been investigated [111,112,113]. Botanicals are commonly utilised as repellents, insecticides, fungicides, and rodent control agents because some plants are known to have broad pesticidal activities [14,114]. Essential oils are complex mixtures of 20 to 60 chemical components, including phenols, sesquiterpenes, and monoterpenes, as reported by Bakkali et al. [115].

Some plant families, such as Rutaceae, Umbelliferae, Myrtaceae, and Labiatae, contain large amounts of chemicals. Together, these substances give the plant’s leaves, flowers, fruits, seeds, bark, and rhizomes their unique flavours and scents [116]. Using essential oils to preserve crops offers several advantages, including low toxicity to mammals, environmental non-persistence, compatibility with biological control agents, and, in some cases, exemption from regulatory authorisation [117,118]. Grain infestations can be controlled with crude oil and leaf powder derived from plant extracts [41]. Numerous essential oils from various spices have been found to possess insecticide properties [119]. In addition to their insecticidal actions, plant oils have virucidal, antifungal, anti-feedant, and reproductive-inhibiting qualities [120].

The use of chemical structures gives a ground for the correlation between phytochemical composition and biological activity to supplement the previous debates on mechanisms of action and formulation strategies.

## 9. Phytochemical

### 9.1. Saponins

Plants contain glycosides called saponins, which are made up of sugar and sapogenin moieties. They are separated into two groups based on the type of aglycone: triterpenoids and steroidal saponins. Saponins comprise compounds such as tirucallane, oleanane, and dammarane [102]. Saponins, also known as surface-active compounds, are abundant in the plant kingdom [121]. Saponins, such as five-ring and four-ring triterpene saponins, have been shown to possess cardioprotective potential through various mechanisms, including regulating energy metabolism, maintaining calcium homeostasis, and mitigating oxidative stress and inflammation [122]. The saponin group functions as a detergent, causing cell membrane rupture, cell death, and ultimately leading to the demise of insect pests. Saponins’ ability to kill insects is mediated by their interaction with cholesterol, which prevents ecdys from synthesising steroids [123]. The majority of legumes have insecticidal (deterrent or repellent) properties due to their saponin content. Increased mortality, decreased food intake, weight loss, developmental retardation, and decreased reproduction are the most often noted impacts of saponins on pests [123].

### 9.2. Alkaloids

Plants produce a class of compounds containing nitrogen called alkaloids in response to their biotic or abiotic environments. As a result, alkaloids exhibit a wide range of structures and possess extraordinary biological functions [110,124]. Several plant families, such as the Compositae, Leguminosae, and Ranunculaceae, contain alkaloids, which are secondary plant metabolites with physiological functions [125]. Nicotine is one of the first chemicals used as a pesticide and the oldest alkaloid utilised in agriculture [125,126]. However, the utilisation of nicotine has reduced due to its toxicity level to humans and animals [125]. Alkaloids are considered anti-nutrients due to their impact on the neurological system, including disruptions or abnormal increases in electrochemical transmission [124,127]. Large doses of tropane alkaloids, for example, would cause a rapid heartbeat, immobility, or, in the worst case, mortality. Glycoalkaloids significantly inhibit cholinesterase, resulting in symptoms similar to those of neurological diseases [127]. Alkaloids can be utilised for both medicinal and recreational purposes and have a proven medical usefulness. Since the majority of them are highly poisonous and bitter, plants may utilise them as protective mechanisms against herbivores, microbial infections, and invertebrate pest infestations [122,128]. In insects, alkaloids can interfere with nerve transmission by disrupting the cytoskeleton and cell membrane, which leads to cell collapse and leakage. They also affect AChE receptors in the nervous system, control hormone activity, and produce toxicity, interfering with important cellular and physiological processes [123,129]. Alkaloids cause a bitter taste in humans, but they can also be unpleasant or a feeding stimulant for certain insects [123,130,131].

### 9.3. Tannins

Tannins are highly specialised phenolic compounds found in the roots, bark, wood, leaves, and fruit, primarily in the lysosomes. Their molecular weights range from 500 Da to over 3000 Da [132,133,134]. Plant defence mechanisms against insects, birds, and mammalian herbivores have been linked to them. Based on chemical composition and characteristics, tannins are grouped into condensed tannins, phlorotannins and hydrolysable tannins [134,135,136]. Exfoliants, free radical scavengers, gastrointestinal tumour inhibitors, antibacterials, diuretics, anti-inflammatory agents, and hemostatic treatments are all applications for phytoconstituents containing tannin. Tannins are used as a clarifying agent in food items such as wine, beer and fruit juices [133,134].

### 9.4. Flavonoids

Flavonoids are one of the primary forms of polyphenols, and they are mostly found in wine, stems, fruits, tea, cereals, seeds, vegetables, flowers and nuts. Their capacity to alter cell signalling pathways and their antioxidant qualities are well-known [100,137,138]. Among the various properties of flavonoids are their anticancer, antimicrobial, anti-inflammatory, anti-proliferative, anti-angiogenic, analgesic, antiviral, and antioxidant effects [101,137,139]. Based on chemical structure, classifications of flavonoids include flavonones, flavanols, flavones, flavonols, anthocyanidins and isoflavones [102,140,141]. Flavonoids serve a range of purposes in plants, including regulating cell growth, attracting pollinating insects, and providing defence against biotic and abiotic challenges [142]. Flavonoids are secondary plant metabolites that have attractive qualities and are found in large quantities, responsible for colour, scent and flavour in seeds, plants, and fruit. For example, neochamaejasmin A, one of *Stellera chamaejasme’s* active components, causes contact stomach toxicity in insects [125,142].

### 9.5. Phenols

Plant defence against pathogenic diseases and insect herbivory in fruits and vegetables is greatly aided by phenolic compounds. Phenols have also been intimately associated with plants’ chemical defence against higher herbivores [143,144,145]. Phenols may affect the insect’s endocrine system or have antimicrobial properties [123].

### 9.6. Terpenoids

Terpenes are naturally occurring hydrocarbons and the primary class of secondary metabolites involved in plant defence mechanisms [123]. This category is present in all plants, which have more than 22,000 compounds [123]. The most common class of secondary metabolites in plants is terpenoids, which are usually present in the vegetative tissues, flowers, and roots [110]. Several terpenoid compounds possess antifungal, chemotherapeutic, anti-hyperglycaemic, antispasmodic, immunomodulatory, antiallergenic, antiviral, antimicrobial, antiparasitic, and anti-inflammatory properties [127,146]. Terpenes are important for plant defence against invading plants and herbivores, chemical signalling, interaction, defence against photooxidation, plant–environment mediation, thermoprotection, and pollinator attractiveness [146]. Terpenes are the main class of compounds found in essential oils. Although there are other varieties of terpenes, monoterpenes and sesquiterpenes are the most significant in essential oils; these two chemical groups are responsible for their distinctive scent [101,147].

## 10. Mechanisms of Action

Modes of action of metabolites can differ, particularly when a complex mixture of compounds is responsible for the activity. These compounds may be poisonous or repulsive to the target organisms, resulting in developmental abnormalities such as diminished growth, altered behaviour, and sterility [148]. Terpenes, sesquiterpenes, aldehydes, ketones, and phenolic compounds are all abundant in essential oils. These elements impact insect nervous systems and behaviour in addition to disrupting cell membranes, preventing spore germination, altering enzyme function, and causing oxidative stress in fungal infections [117]. Essential oils and plant extracts can pass through insect cuticles because they are very lipophilic [116]. Certain botanical formulations can cause contact, gastrointestinal, and respiratory poisoning. They might function as repellents, antifeedants, or inhibitors of phenology [149]. Rotenone is a powerful inhibitor of cellular respiration that causes damage to both muscle cells and nerve cells. Monoterpenes from essential oils inhibit cholinesterase, and in nerve axons, pyrethrins block voltage-gated sodium channels. Insect populations are also significantly reduced due to certain plant substances that interfere with normal growth and moulting processes [116].

Transcriptome analysis is a useful tool in the development of sophisticated plant protection techniques because it makes it easier to identify functional genes linked to resistance mechanisms in host plants and clarifies the molecular interactions involved in host–pathogen dynamics [150]. Plants employ a complex network of signalling channels, transcriptional modifications, and biochemical reactions as part of their molecular and physiological defensive mechanisms. Important signalling molecules that regulate various aspects of plant defence include salicylic acid (SA), jasmonic acid (JA), ethylene (ET), and other compounds [151,152]. Abscisic acid (ABA), a plant hormone, is essential for regulating many growth- and development-related physiological processes and for mediating plants’ adaptive responses to biotic and abiotic stressors [150]. It has been established that plant hormones, including auxins, gibberellins, cytokinins, brassinosteroids, and abscisic acid (ABA), play important roles in defence mechanisms against colonisation attempts and pathogen attacks [152]. Herbivory induces reactive oxygen species (ROS), which have been linked to the regulation of plant defence [153]. Inducible secondary metabolites in plants are emerging as defence regulators to increase the specificity of the signalling networks underlying plant defence responses [153]. Defence priming is commonly associated with induced resistance (IR), including mycorrhiza-induced resistance (MIR), induced systemic resistance (ISR), and systemic acquired resistance (SAR) [154].

In addition to having direct toxic and repellent effects, plant-derived compounds are important in the activation of plant defence systems, thereby facilitating pest and disease control in an indirect manner. There is growing interest in suggesting that these compounds may be used as elicitors to activate complex immune responses in plants.

Plant-derived bioactives have the potential to stimulate major defence pathways, such as PAMP-induced immunity (PTI), effector-induced immunity (ETI) and systemic acquired resistance (SAR), resulting in increased resistance to a broad spectrum of pathogens. These reactions are signalled by complex signalling pathways of phytohormones like salicylic acid, jasmonic acid, abscisic acid and auxins, which mediate the expression of defence-related genes and synthesis of protective metabolites.

**Figure 11:**
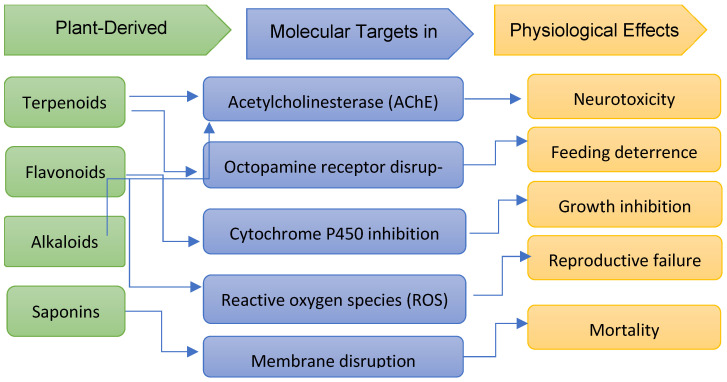
Schematic representation of the mechanisms of action of plant-derived phytochemicals.

Nevertheless, these mechanisms have not been widely incorporated into the study of biopesticides, although there is an increase in interest in these mechanisms. The majority of research remains focused on direct pesticidal effects, and comparatively limited research has been performed looking at the effect of plant-based compounds in the modulation of host plant immunity. This disequilibrium can be viewed as a major disparity in the existing research and restricts the complete utilisation of these compounds in sustainable agriculture. Figure 11 shows the schematic representation of the mechanisms of action of plant-derived phytochemicals.

## 11. Pests and Diseases of Maize Targeted by Medicinal Plants

Farmers in sub-Saharan Africa have investigated locally accessible traditional methods for managing pests and crop diseases. It was reported that 177 plants from 54 families are utilised to manage agricultural diseases and pests. The Fabaceae, Asteraceae, Solanaceae, Euphorbiaceae, Lamiaceae, and Malvaceae are the plant families with the greatest number of species used to manage pests and crop diseases; together, they comprise approximately 49% of the plant species mentioned. The remaining 48 families comprise 51% of the plant species used to manage crop pests and diseases [14].

Four plant powders were evaluated for weevil control in maize stored in Oaxaca, Mexico: epazote (*Chenopodium ambrosioides* L.), oregano (*Origanum vulgare*), hierba santa (*Piper auritum*), and laurel (*Laurus nobilis*). The species with the highest mortality and repellency were Hierba Santa and epazote. Numerous secondary metabolites in these plant species under investigation are responsible for the insecticidal and repellent effects observed, although their metabolites were not determined in this research [11]. However, Kasali et al. documented that through the use of essential oils, approximately 330 compounds, including various isomers, have been identified in *C. ambrosioides* in its different parts. Monoterpenes make up the majority of these compounds, including ketones, esters, glycosides, aliphatic acids, flavonoids, aromatic hydrocarbons, and carbohydrates, among others [155].

Research by Cortese et al. utilised botanical extracts of *Schinus terebinthifolius*, *Ludwigia sericea*, *Ludwigia nervosa*, *Ludwigia longifolia*, and *Ludwigia tomentosa* as plant-based insecticides for maize weevil, following assessments of their repellency potential and the decrease in offspring emergence. Even at 48 h after treatment, all of the chosen plant extracts showed repellent activity against *Sitophilus zeamais*. The presence of the maize weevil was considerably inhibited by the *L. nervosa* aqueous extract [156]. Suleiman et al., Suleiman et al., and Cortese et al. reported that many *Schinus species* contain active substances, such as saponins, phenolic compounds, alkaloids, tannins, and flavonoids, that can interfere with olfactory receptors, preventing insects from detecting their hosts [81,156,157]. Fernandes & Favero also reported on the effectiveness of using *S. molle* L. essential oil against *S. zeamais*. This plant’s oil exhibits a contact and repellent insecticide effect, which can have a variety of negative effects on the insects, including inhibition of oviposition, growth, and feeding, as well as morphogenetic changes, disruption of the hormonal system, mortality in both adult and immature stages, and alteration of sexual behaviour [158].

Seepe et al. recorded the antifungal activity of extracts from several plant species against the phytopathogenic *Fusarium species*. Various solvent extracts from 47 plant species across 30 families were recorded. The Combretaceae (four species), Euphorbiaceae (three species), Fabaceae (four species), and Solanaceae (six species) are families with a high frequency of examined species that are resistant to Fusarium pathogens. *Solanum aculeastrum*, *Nicotiana glauca*, *Solanum seaforthianum*, and *Solanum mauritianum* are some of the Solanaceae family plants that were assessed. Strong in vitro activity against nine Fusarium species was demonstrated by the leaf extracts of these plants (minimum inhibitory doses < 1.0 mg/mL) [159].

## 12. Advances in Biopesticide Formulation and Application

Utilising nanoparticles and nanotechnology in biological products is a relatively recent advancement. It is anticipated that this technological development would increase the efficacy of botanical biocontrol techniques. Future studies may also concentrate on the chemical analysis of plant-based pesticides and fungicides. This involves identifying novel bioactive substances and evaluating their effectiveness in controlling pests and agricultural diseases [117,160,161].

One significant development in food processing and agricultural protection is the synthesis of nanoparticles and pesticide delivery devices. With an emphasis on efficacy, safety, specificity, and environmental impact, nanotechnologies are being applied to enhance food items through nanocoatings, nanofoods, nanoencapsulation, and nanoemulsions [117,162,163]. Recent developments in nanotechnology and nanoscience have brought about significant advances in agricultural pest management. The use of artificial nanomaterials as key components in nano-based pesticide formulations has attracted a lot of attention because of their effectiveness in managing insects and protecting crops. Compared to conventional pesticides, nano-pesticides offer superior environmental performance by boosting target specificity and minimising non-target impacts, while also minimising residue buildup in soil and water systems [164,165].

Additional advantages of nano-pesticides include improved foliar adherence, larger crop yields, and higher-quality output. When used properly, these benefits have the potential to greatly increase agricultural output and encourage environmentally friendly farming methods [164,166]. By integrating nanotechnology and biopesticides, new formulations have been developed to efficiently manage pests while reducing the hazards associated with chemical pesticides and overcoming the limitations of biopesticides [166]. Among the improved precision targeting methods and spray technologies that have been developed are automated targeting systems, robotics, automation technologies, air-assisted sprayers, intelligent spray systems, thermal fogging, controlled droplet application, variable-rate technology, and aerial application [167]. Among the enhanced precision targeting methods and spray technologies that have been developed are systems that optimise droplet size and spray characteristics to reduce drift and improve deposition, increase pesticide use efficiency, and lessen environmental contamination [30,168].

However, according to reports, most nanoparticles may be harmful in specific quantities and affect crop yield by altering the morphological, anatomical, biochemical, genetic, and physiological characteristics of the crops. Size, shape, chemical composition, reactivity, and surface charge are examples of physicochemical properties that can influence the ability of seeds to absorb and transport nanoparticles [169,170,171]. Biopesticides must be carefully formulated to maintain their biological viability because they are sensitive to environmental influences and storage conditions. Some of the challenges related to the development of biopesticides include ensuring the product’s long-term viability and efficacy, ease of manufacture and usage, market stability, and maintaining product stability throughout storage and transportation [66,167]. Several issues, including inadequate formulation, production challenges, and susceptibility to environmental pressures, have been cited as contributing to their poor commercial performance [66,167,172]. Compared to chemical insecticides, which often require more frequent treatments, biopesticides typically have a shorter half-life [66]. Obtaining regulatory approval can be a challenging and time-consuming process [66]. Farmers may face difficulties if biopesticides are not readily available. It can also be difficult to determine whether they successfully support integrated pest management. The consistent effectiveness of biopesticides may be impacted by a lack of standardisation and quality control procedures [66,167].

Nanotechnology offers sophisticated delivery methods that can improve these biopesticides’ stability, solubility, and focused activity, ultimately resulting in more successful pest management strategies [173,174]. Nanotechnology lowers the required dosages and extends the duration of the pesticide action by increasing the bioavailability, stability, and efficacy of bioactive compounds [166]. Nanopesticide formulations improve water solubility, bioavailability, and protect agrochemicals from environmental deterioration, transforming the management of weeds, insects, and diseases in crops [175,176]. Pesticides are nanoencapsulated for various reasons, including enhanced activity from improved interaction with the pathogen, insects, weeds, and other pests, and loss of efficacy from evaporation, degradation, and leaching [175]. The size and structure of nano-biopesticides are different from those of traditional biopesticides. They range in size from 1 to 100 nm. They are particles containing active components or engineered structures that reduce, lessen, or stop pest-related damage [173,174]. In addition to protecting bioactive substances, nanoencapsulation and nanocoating also improve transport through natural barriers, increase bioavailability, and offer controlled distribution to a specific area [177].

The recent developments in the science of formulations have made nanotechnology a revolutionary method in improving the functioning of plant-based biopesticides. Nanoencapsulation, nanoemulsions and polymer delivery systems have been extensively developed to overcome the major constraints (poor stability, low solubility and quick environmental degradation) of plant-derived compounds. But, despite these benefits, nanotechnology has not been applied in biopesticides without any restrictions. Scalability of nanoformulation processes is also a major issue since most methods are best scaled in the laboratory but are challenging to scale up to cost-efficient industrial applications. Moreover, most of the studies that claim to have improved efficacy of nanoformulated biopesticides are done under controlled experimental conditions, and there are few studies that have been validated to have efficacy in field situations. Therefore, although nanotechnology is a promising tool to resolve the most important formulation issues, it should be applied in practice with additional studies that should be devoted to cost-cutting, mass production, environmental friendliness, and field testing. A combination of nanotechnology and ethnobotanical information and the use of phytochemical standardisation has the potential to offer a more sturdy route toward the creation of effective and scalable plant-based biopesticides.

## 13. Research Gaps and Future Prospects

A critical review of the studies indicates that although plant-derived compounds have good pesticidal properties, their limitations to practical use are associated with differences in phytochemical composition, absence of standardised formulations, and unreliable performance in the field. Moreover, direct toxicity impacts are the main focus of most studies, and little has been done to investigate indirect effects, including induced plant resistance and hormonal signalling. This discrepancy points to a critical gap in the research and the necessity of uniting efforts between phytochemistry, molecular plant biology and formulation science.

Most studies either record the traditional uses of plants or assess their effectiveness in isolation in the laboratory. Few studies have systematically linked ethnobotanical knowledge to bioassay results and phytochemical validation. Because different studies employ varying solvents, concentrations, and extraction methods, it is challenging to compare the results. Most of the proof is still generated under laboratory conditions. Few studies have evaluated plant extracts or essential oils in real-world farming environments or examined the stability and persistence of formulations under storage and environmental exposure conditions. Although some phytochemicals have been identified, little is known regarding their target selectivity, synergistic effects, and mechanisms of action. There has been little consideration given to the safety of plant-based formulations for non-target organisms, including humans, beneficial insects, and soil microorganisms. Moreover, the integration of indirect processes like induced plant immunity and defence priming signals a paradigm shift in the perspective of plant-derived compounds as only toxic substances to the perception of them as regulators of plant defence systems. This two-fold action creates new possibilities to create more sustainable and resilient pest management practices. In many African nations, there are currently no explicit laws or frameworks in place to support the marketing, quality assurance, and registration of locally produced plant-based biopesticides. Future studies, however, should focus on developing a comprehensive framework that integrates phytochemical profiling, application of ethnobotanical knowledge, and large-scale field-based validation to produce standardised, eco-friendly, and commercially viable plant-based biopesticides for maize protection in Africa.

## 14. Conclusions

In Africa, medicinal plants represent a promising agent for managing diseases and pests that affect maize. Because these plants contain a variety of phytochemicals with antibacterial and pesticidal properties, they offer potential as a sustainable alternative. However, additional field validation and safety evaluation are required. *Zanthoxylum zanthoxyloides*, *Azadirachta indica*, *Carica papaya*, *Moringa oleifera*, and *Ficus exasperata* are some of the plants proven to be effective against crop pests and diseases. The effective compounds include flavonoids, tannins, saponins, phenols and alkaloids. Due to problems such as poor formulation techniques, a lack of standardisation, and limited research on active chemicals, the true potential of medicinal plants for controlling diseases and pests remains largely unexplored, despite their demonstrated efficacy. Despite the promising results in the laboratory, there is still limited field application, and more research is needed to assess effectiveness in actual farming conditions and possible effects on non-target organisms. The review suggests expanding research on the separation and characterisation of active components, improving extraction and formulation methods, and fusing traditional ethnobotanical knowledge with contemporary technologies. Enacting laws that facilitate the production and marketing of plant-based biopesticides will enhance environmental protection and promote environmentally friendly farming methods.

## Figures and Tables

**Table 1:** Medicinal plants traditionally used to control *Sitophilus zeamias* across several studies.

Scientific Names of Plants	Family	Part Used	Solvent Used	Mode of Action	Major Active Ingredients	Key Findings	References
*Euphorbia balsamifera*, *Lawsonia inermis*, *Mitracarpus hirtus*, and *Senna obtusifolia*	Euphorbiaceae, Lythraceae, Rubiaceae, Fabaceae	Leaves	Powder, methanolic, ethanolic, and aqueous	Repellent	N/A	*Senna obtusifolia* was the least repellent of the botanicals, while *Euphorbia balsamifera* was shown to have a better repellency than permethrin powder.	[81]
*Aristolochia ringens* (Vahl), *Allium sativum* (L), *Ficus exasperata* (L), and *Garcinia kola* (H)	Aristolochiaceae, Liliaceae, Moraceae, Guttiferae	Roots, bark, bulbs, leaves, and seeds	Petroleum ether	Protectant	N/A	*A. sativum* and *A. ringens* showed some promise as repellents, food poisons, contact poisons, and antifeedants.	[82]
*Cleome monophyla*	Capparidaceae	Shrub	Essential oil	Repellent	Terpenolene, 1-a-terpeneol, pentacosane, (a + 3)-humulene, phytol, 2-dodecanone	The most repellent components against *Sitophilus zeamais* were l-a-terpeneol and 2-dodecanone.	[83]
*Calpurnia aurea* (Ait.) Benth and *Milletia ferruginea* (Hochst.) Baker	Fabaceae	Leaves	Distilled water, acetone, and ethanol	Protectant	N/A	At a rate of 10% and 15%, all of the evaluated polar solvent extracts of *C. aurea* and *M. ferruginea* were effective in defending maize grains against attacks by maize weevils.	[84]
*Calpurnia aurea*	Fabaceae	Leaves	Ethanol, acetone, methanol, distilled water, chloroform, n-hexane	Repellent	N/A	Three days after treatment, a 10% dosage of the polar solvent extract from *Calpurnia aurea* leaves exhibited 100% weevil repellency.	[85]
*Aframomum melegueta* and *Zingiber officinale*	Zingiberaceae	Seed and rhizome	Distilled water, hexane, and diethyl ether	Repellent	(*S*)-2-heptanol, (*S*)-2-heptyl acetate, (*R*)-linalool, and 1,8-cineole	For *A. melegueta*, the repellent activity was accounted for by a synthetic blend of (*S*)-2-heptanol, (*S*)-2-heptyl acetate, and (R)-linalool and for *Z. officinale* by a synthetic blend of 1,8-cineole, neral, and geranial.	[86]
*Artemisia capillaris* and *Artemisia mongolica*	Asteraceae	Aerial parts	Essential oil	Contact and fumigant	1,8-cineole, germacrene D, and camphor. α-pinene, germacrene D, and γ-terpinene	The two essential oils were ten times less toxic to the maize weevil than the commercial fumigant MeBr, but they still had substantial fumigant toxicity against adult weevils. Additionally, the two essential oils demonstrated contact toxicity to maize weevils.	[87]
*Zanthoxylum zanthoxyloides*, *Aristolochia ringens*, *Garcinia kola*, *Morinda lucida*, *Euphorbia hirta*, *Croton zambesicus*, *Colocasia esculenta*, *Ficus exasperata*, and *Tetrapleura tetraptera*	Rutaceae, Aristolochiaceae, Clusiaceae, Rubiaceae, Euphorbiaceae, Araceae, Moraceae, Fabaceae	Roots, seeds, stem bark, shoot, leaves, and fruits	Powder	N/A	N/A	*Zanthoxylum zanthoxyloides* powder had the greatest effect on weevil mortality, reaching 100% in 72 h at 5% (*wt*/*wt*).	[88]
*Momordica charantia*, *Annona muricata*, *Averrhoa bilimbi*, *Ocimum Selloi*, *Lantana camara*, *Ailanthus altissima*, *Eugenia caryophyllata*, *Azardirachta indica*, *Cassia alata*, *Ricinus communis*	Cucurbitaceae, Annonaceae, Oxalidaceae, Lamiaceae, Verbenaceae, Simaroubaceae, Myrtaceae, Meliaceae, Fabaceae, Euphorbiaceae	Leaves	Methanol	Contact and repellent	Flavonoid, alkaloid, tannins, and saponins	The methanol extract of *A. muricata* showed the highest mortality, with 100% mortality within 7 days. *O. selloi* showed maximum (57.80 ± 21.2) repellency.	[89]
*Curcuma longa* and *Piper guineense*	Zingiberaceae, Piperaceae	Rhizome, seeds	Powder and ethanol	Protectant	N/A	*P. guineense* powder was the most effective; the extracts were more toxic than the powders.	[90]
*Daucus setifolius*	Apiaceae	Aerial parts	Essential oil	Repellent	Sabinene, selinene, terpinen-4-ol	After 120 min of exposure, the volatile oil showed greater repellency to *Sitophilus zeamais.*	[91]

N/A = Not available (not reported in the literature).

**Table 2:** Some medicinal plants traditionally used to control pathogens/diseases of maize.

Scientific Names of Plants	Family	Part Used	Target Pathogen/Diseases	Solvents	Major Active Ingredients	Key Findings	References
*Withania somnifera*, *Combretum molle*, *Combretum erythrophyllum*, *Quercus acutissima*, *Solanum mauritianum*, *Melia azedarach*, *Lantana camara* and *Nicotiana glauca*	Solanaceae, Combretaceae, Fagaceae, Solanaceae, Meliaceae, Verbenaceae Solanaceae	Leaves	*Fusarium* pathogens (*F. proliferatum*, *F. oxysporum*, *F. subglutinans*, *F. verticilloides*, *F. semitectum*, *F. chlamydosporum*, *F. solani*, *F. equisite*, and *F. graminearum*)	water, ethyl acetate, or acetone	N/A	This study concluded that plant extracts could prevent Fusarium diseases without having a negative impact on plant growth or maize seed germination.	[92]
*Justicia specie*	Acanthaceae	Stem and leaf	*Fusarium graminearum*/Ear rot	dichloro-methane, methanol, and ethyl acetate	lignans hino-kinin, isohibalactone, and savinin	Only the *J. xylosteoides* leaf and stem extracts showed inhibitory activity, with the dichloromethane leaf extract having the highest level of effectiveness.	[93]
*Eucalyptus tereticornis* Sm., *Ammi visnaga* (L.) Lam., *Azadirachta indica* A. Juss., *Rheum Palmatum* L., and *Adansonia digitata* L.	Myrtaceae Apiaceae Meliaceae Polygonaceae Malvaceae	Leaves, seeds, fruits, and roots	*Rhizoctonia* root rot (*Rhizoctonia solani)*	Methanol	*Ammi visnaga*: 8-methoxyp-soralen, glycerin, 3-piperidin-1-yl-1-(4-piperidin-1-yl-phenyl)-pyrrolidine-2,5-dione, 2-monopalmitin, palmitic acid, khellin, xanthyletin, cyclopenta[c]pyran-7-carboxaldehyde, oleic Acid, and 2-(1-Hydroxy-1-methylethyl)-2,3-dihydrofuro3,2 chromen-7-one	This study showed how *Ammi visnaga* methanol extracts can activate the maize immune system against *Rhizoctonia solani.*	[94]
*Anthemis nobilis*, *Cinnamomum verum*, *Lavandula stoechas*, *Malva sylvestris*, *Mentha piperita*, and *Allium sativum*	Asteraceae, Lauraceae, Lamiaceae, Malvaceae, Lamiaceae, and Amaryllidaceae	Dried leaves, bulb	*Fusarium culmorum*, *Aspergillus candidus*, *Penicillium sp*. and *Aspergillus niger*,	Aqueous extracts	N/A	The growth of the examined fungi was inhibited by the more concentrated extracts of chamomile and malva, with malva having the highest level of efficiency.	[95]
*Veronica amygdalina* Siam, *Chromolaena odorata*, *Azadirachta indica*, and a combination of all the plants *Waltheria indica* L. *Senna tora* (L.) Roxb. *Vernonia amygdalina* Delile	Asteraceae, Meliaceae Malvaceae Fabaceae Asteraceae	Leaves Root, stem, leaf, seed, and flower	*Curvularia* leaf spot (*Curvularia lunata*) Fungal ear rot (*Fusarium graminearum*)	Sterile distilled water Aqueous extracts	N/A N/A	Among all plant extracts, neem extract proved the most effective in controlling Curvularia leaf spot. The findings showed that all the plant extracts under investigation had an inhibitory effect on the conidial growth of *F. graminearum*.	[96,97]

N/A = Not available (not reported in the literature).

## Data Availability

No new data were created or analysed in this study.

## References

[1] Awata L.A., Tongoona P., Danquah E., Ifie B.E., Suresh L., Jumbo M., Marchelo-D’ragga P.W., Sitonik C. (2019). Understanding Tropical Maize (*Zea mays* L.): The Major Monocot in Modernisation and Sustainability of Agriculture in Sub-Saharan Africa. Int. J. Adv. Agric. Res..

[2] Erenstein O., Jaleta M., Sonder K., Mottaleb K., Prasanna B.M. (2022). Global Maize Production, Consumption and Trade: Trends and R&D Implications. Food Secur..

[3] Yadesa L., Diro D. (2023). Nutritional and Specialty Maize Production, Consumption, and Promising Impact on Ethiopia’s Food and Nutrition Security: A Review. EAS J. Nutr. Food Sci..

[4] Shiferaw B., Prasanna B.M., Hellin J., Bänziger M. (2011). Crops That Feed the World 6. Past Successes and Future Challenges to the Role Played by Maize in Global Food Security. Food Secur..

[5] Pingali P. (2007). Westernisation of Asian Diets and the Transformation of Food Systems: Implications for Research and Policy. Food Policy.

[6] Makkar H. (2018). Feed Demand Landscape and Implications of Food-Not Feed Strategy for Food Security and Climate Change. Animal.

[7] Simanjuntak C., Gaiser T., Ahrends H.E., Ceglar A., Singh M., Ewert F., Srivastava A.K. (2023). Impact of Climate Extreme Events and Their Causality on Maize Yield in South Africa. Sci. Rep..

[8] Jombo S., Abd Elbasit M.A., Zhang C., Gumbo A.D. (2025). Opportunities and Challenges for Monitoring Maize Production in Sub-Saharan Africa: A Comprehensive Bibliometric Analysis of Remote Sensing Applications. Sci. Afr..

[9] Niazi P., Hejran A.B. (2025). Biopesticides for Sustainable Agriculture: Formulations, Mechanisms, Regulations, and Market Trends. AgroTech-Food Sci. Technol. Environ..

[10] Masese C., Odira M., Marwa C., Masese D., Ponoth P., Gikonyo A. (2025). Exploring the Potential of Plant-Based Biopesticides for Sustainable Crop Protection in Mwea, Kirinyaga County, Kenya. Scholast. Agric..

[11] Peña-Flores C., Zapién-Martínez A., Sánchez-Cruz G., Reyes-Velasco L., Segura-Salvador A., Vargas-Arzola J., Hernández-Osorio L.A., Torres-Aguilar H., Bernardino-Hernández H.U. (2025). Insecticidal and Repellent Activity of Plant Powders on the Weevil (*Sitophilus zeamais*) in Stored Corn Grains in a Rural Community of Oaxaca, Mexico. Insects.

[12] Asibe F.A., Ngegba P.M., Mugehu E., Afolabi C.G. (2023). Status and Management Strategies of Major Insect Pests and Fungal Diseases of Maize in Africa: A Review. Afr. J. Agric. Res..

[13] Pavela R. (2016). History, Presence and Perspective of Using Plant Extracts as Commercial Botanical Insecticides and Farm Products for Protection against Insects–a Review. Plant Prot. Sci..

[14] Shai K.N., Chakale M.V., Materechera S.A., Amoo S.O., Aremu A.O. (2024). Utilisation of Botanicals for the Management of Pests and Diseases Affecting Crops in Sub-Saharan Africa: A Review. J. Nat. Pestic. Res..

[15] Shamsuddeen R., Mathew T., Haladu M., Ahmad K., Adam A., Abubakar M. (2024). Recent Advances in Biopesticides: A Review of Efficacy and Environmental Impact. Afr. J. Biochem. Mol. Biol. Res..

[16] Anjaneyulu B., Chauhan V., Mittal C., Afshari M. (2024). Innovative Nanocarrier Systems: A Comprehensive Exploration of Recent Developments in Nano-Biopesticide Formulations. J. Environ. Chem. Eng..

[17] Isman M.B. (2020). Botanical Insecticides in the Twenty-First Century—Fulfilling Their Promise?. Annu. Rev. Entomol..

[18] Labonete H.J.P., Jimenez E.A., Torres M.A.J., Demayo C.G. (2025). Ethnobotanical and Phytochemical Insights on Insecticidal Plants in the Philippines for Sustainable Crop Protection: A Systematic Review and Network Analysis. Int. J. Agric. Biosci..

[19] Silvie P.J., Martin P., Huchard M., Keip P., Gutierrez A., Sarter S. (2021). Prototyping a Knowledge-Based System to Identify Botanical Extracts for Plant Health in Sub-Saharan Africa. Plants.

[20] Aisha K., Visakh N.U., Pathrose B., Mori N., Baeshen R.S., Shawer R. (2024). Extraction, Chemical Composition and Insecticidal Activities of *Lantana camara* Linn. Leaf Essential Oils against *Tribolium castaneum*, *Lasioderma serricorne* and *Callosobruchus chinensis*. Molecules.

[21] Benjamin J., Idowu O., Babalola O.K., Oziegbe E.V., Oyedokun D.O., Akinyemi A.M., Adebayo A. (2024). Cereal Production in Africa: The Threat of Certain Pests and Weeds in a Changing Climate—A Review. Agric. Food Secur..

[22] Njeru F., Wambua A., Muge E., Haesaert G., Gettemans J., Misinzo G. (2023). Major Biotic Stresses Affecting Maize Production in Kenya and Their Implications for Food Security. PeerJ.

[23] Lobulu J., Shimelis H., Laing M., Mushongi A.A. (2019). Maize Production Constraints, Traits Preference and Current Striga Control Options in Western Tanzania: Farmers’ Consultation and Implications for Breeding. Acta Agric. Scand. Sect. B—Soil Plant Sci..

[24] Agbodzavu K.M., Nanga Nanga S., Abang A.F., Fotso-Kuate A., Bamba Z., Masso C., Fiaboe K.K.M. (2024). Impact of *Spodoptera frugiperda* (*Lepidoptera: Noctuidae*), on Maize Yield in Humid Tropical Zones of Central Africa. J. Econ. Entomol..

[25] Taddele A., Azerefegne F., Beyene Y. (2023). Crop Injury and Yield Losses in Maize by the African Maize Stem Borer, *Busseola fusca* (Fuller) (*Lepidoptera: Noctuidae*) in Southern Ethiopia. Int. J. Pest. Manag..

[26] Abbas A., Ullah F., Hafeez M., Han X., Dara M.Z.N., Gul H., Zhao C.R. (2022). Biological Control of Fall Armyworm, *Spodoptera frugiperda*. Agronomy.

[27] Mohamed H.O., Dahi H.F., Awad A.A., Gamil W.E., Fahmy B.F. (2023). Damage Symptoms, Development, and Reproductive Performance of the Fall Armyworm, *Spodoptera frugiperda* (JE Smith) (Lepidoptera: Noctuidae) on Fodder Maize and Cob. Acad. Biol..

[28] Makgoba M.C., Tshikhudo P.P., Nnzeru L.R., Makhado R.A. (2021). Impact of Fall Armyworm (*Spodoptera frugiperda*) (JE Smith) on Small-Scale Maize Farmers and Its Control Strategies in the Limpopo Province, South Africa. Jàmbá J. Disaster Risk Stud..

[29] Pfordt A., Paulus S. (2025). A Review on Detection and Differentiation of Maize Diseases and Pests by Imaging Sensors. J. Plant Dis. Prot..

[30] Khan J., Gul P., Liu K. (2024). Grains in a Modern Time: A Comprehensive Review of Compositions and Understanding Their Role in Type 2 Diabetes and Cancer. Foods.

[31] Duguma H.T. (2020). Indigenous Knowledge of Farmer on Grain Storage and Management Practice in Ethiopia. Food Sci. Nutr. Technol..

[32] Bisheko M.J., Rejikumar G. (2023). Major Barriers to Adoption of Improved Postharvest Technologies among Smallholder Farmers in Sub-Saharan Africa and South Asia: A Systematic Literature Review. World Dev. Sustain..

[33] Garcia-Cela E., Kiaitsi E., Sulyok M., Krska R., Medina A., Petit Damico I., Magan N. (2019). Influence of Storage Environment on Maize Grain: CO_2_ Production, Dry Matter Losses and Aflatoxins Contamination. Food Addit. Contam. Part A.

[34] Gumede B.C., Kuria S.K. (2025). Postharvest Practices and Farmers’ Knowledge in Managing Maize Pests in the Eastern Cape Province, South Africa. Insects.

[35] Sola P., Mvumi B., Ogendo J., Mponda O., Kamanula J., Nyirenda S., Belmain S., Stevenson P. (2014). Botanical Pesticide Production, Trade and Regulatory Mechanisms in Sub-Saharan Africa: Making a Case for Plant-Based Pesticidal Products: P. Sola et al. Food Secur..

[36] Berhe M., Subramanyam B., Chichaybelu M., Demissie G., Abay F., Harvey J. (2022). Post-Harvest Insect Pests and Their Management Practices for Major Food and Export Crops in East Africa: An Ethiopian Case Study. Insects.

[37] Chidege M.Y., Venkataramana P.B., Ndakidemi P.A. (2024). Enhancing Food Grains Storage Systems through Insect Pest Detection and Control Measures for Maize and Beans: Ensuring Food Security Post-COVID-19 Tanzania. Sustainability.

[38] Neupane B.P., Sharma P.N., Aryal S., Shrestha J. (2022). Evaluation of Locally Available Botanicals for the Management of Maize Weevil (*Sitophilus zeamais* Motsch.) in Room Storage Condition. Psyche J. Entomol..

[39] Ndebugri A.A.I., Kugbe J.X., Adu-Acheampong S., Kyerematen R. (2024). Two Plant Extracts Protect Stored Maize against Infestation of *Sitophilus zeamais* in Northern Ghana. J. Nat. Pestic. Res..

[40] Obeng-Ofori D. (2011). Protecting Grain from Insect Pest Infestations in Africa: Producer Perceptions and Practices. Stewart Postharvest Rev..

[41] Ayalew A.A. (2020). Insecticidal Activity of *Lantana camara* Extract Oil on Controlling Maize Grain Weevils. Toxicol. Res. Appl..

[42] Sintim H.O., Ansah K.D. (2023). Effects of Biopesticides Extracted with a Homemade Solvent on Stored Maize Protection. Agric. Trop. Subtrop..

[43] Phokwe O.J., Manganyi M.C. (2023). Medicinal Plants as a Natural Greener Biocontrol Approach to “The Grain Destructor” Maize Weevil (*Sitophilus zeamais*) Motschulsky. Plants.

[44] Kadi H.A.K., Ibrahim A.M., Pendleton B.B., Aboubacar K. (2025). Efficacy of Selected Botanical Powders to Control Maize Weevil, *Sitophilus zeamais* Motschulsky in Stored Sorghum Grain. J. Agric. Chem. Environ..

[45] Wakhungu C. (2023). Loss of Soil Biodiversity through Judicious Use of Synthetic Pesticides; A Case Study of Trans Nzoia County, Kenya. Sci. Rep. Life Sci..

[46] Uwamahoro C., Jo J.-H., Jang S.-I., Jung E.-J., Lee W.-J., Bae J.-W., Kwon W.-S. (2024). Assessing the Risks of Pesticide Exposure: Implications for Endocrine Disruption and Male Fertility. Int. J. Mol. Sci..

[47] Kozuharova E., Pasdaran A., Al Tawaha A.R., Todorova T., Naychov Z., Ionkova I. (2022). Assessment of the Potential of the Invasive Arboreal Plant *Ailanthus altissima* (Simaroubaceae) as an Economically Prospective Source of Natural Pesticides. Diversity.

[48] López-Benítez A., Guevara-Lara A., Domínguez-Crespo M.A., Andraca-Adame J.A., Torres-Huerta A.M. (2024). Concentrations of Organochlorine, Organophosphorus, and Pyrethroid Pesticides in Rivers Worldwide (2014–2024): A Review. Sustainability.

[49] Navarro I., De la Torre A., Sanz P., Abrantes N., Campos I., Alaoui A., Christ F., Alcon F., Contreras J., Glavan M. (2024). Assessing Pesticide Residues Occurrence and Risks in Water Systems: A Pan-European and Argentina Perspective. Water Res..

[50] Gulzar M., Maqsood R., Abbas H., Manzoor M., Suleman M., Bajwa H., Hamza A., Yar S., Zain M., Wadood A. (2024). Use of Insecticides and Their Impact on Viral Diseases in Humans, Animals and Environment. Hosts Viruses.

[51] Yadav I.C., Devi N.L. (2017). Pesticides Classification and Its Impact on Human and Environment. Environ. Sci. Eng..

[52] Abdollahdokht D., Gao Y., Faramarz S., Poustforoosh A., Abbasi M., Asadikaram G., Nematollahi M.H. (2022). Conventional Agrochemicals towards Nano-Biopesticides: An Overview on Recent Advances. Chem. Biol. Technol. Agric..

[53] Ngubane Z., Dzwairo B., Moodley B., Stenström T.A., Sokolova E. (2023). Quantitative Assessment of Human Health Risks from Chemical Pollution in the uMsunduzi River, South Africa. Environ. Sci. Pollut. Res..

[54] Hammad A.M.A., Bashir H.A.A.A., Abdelbagi A.O., Ishag A.E.S.A., Ali M.M.Y., Bashir M.O., Hur J.-H., Laing M.D. (2022). Efficacy of Indigenous Entomopathogenic Fungi for the Control of the Tomato Leafminer *Tuta absoluta* (Meyrick) in Sudan. Int. J. Trop. Insect Sci..

[55] Andersson E., Isgren E. (2021). Gambling in the Garden: Pesticide Use and Risk Exposure in Ugandan Smallholder Farming. J. Rural. Stud..

[56] Ratto F., Bruce T., Chipabika G., Mwamakamba S., Mkandawire R., Khan Z., Mkindi A., Pittchar J., Chidawanyika F., Sallu S.M. (2022). Biological Control Interventions and Botanical Pesticides for Insect Pests of Crops in Sub-Saharan Africa: A Mapping Review. Front. Sustain. Food Syst..

[57] Chebbac K., Benziane Ouaritini Z., Allali A., Tüzün B., Zouirech O., Chalkha M., El Moussaoui A., Lafraxo S., Nafidi H.-A., Bin Jardan Y.A. (2023). Promising Insecticidal Properties of Essential Oils from *Artemisia aragonensis* Lam. and *Artemisia negrei* L. (Asteraceae) by Targeting Gamma-Aminobutyric Acid and Ryanodine Receptor Proteins: In Vitro and in Silico Approaches. Separations.

[58] Evangelou E., Ntritsos G., Chondrogiorgi M., Kavvoura F.K., Hernández A.F., Ntzani E.E., Tzoulaki I. (2016). Exposure to Pesticides and Diabetes: A Systematic Review and Meta-Analysis. Environ. Int..

[59] Curl C.L., Spivak M., Phinney R., Montrose L. (2020). Synthetic Pesticides and Health in Vulnerable Populations: Agricultural Workers. Curr. Environ. Health Rep..

[60] Guerrero Ramírez J.R., Ibarra Muñoz L.A., Balagurusamy N., Frías Ramírez J.E., Alfaro Hernández L., Carrillo Campos J. (2023). Microbiology and Biochemistry of Pesticides Biodegradation. Int. J. Mol. Sci..

[61] Rasool S., Rasool T., Gani K.M. (2022). A Review of Interactions of Pesticides within Various Interfaces of Intrinsic and Organic Residue Amended Soil Environment. Chem. Eng. J. Adv..

[62] Matthew O., Folake A.O., Idu O.M., Philip M.Y. (2023). Beneficial Activities of Ethnobotanical Plants on Selected Systems and Agriculture. J. Med. Plants Stud..

[63] Gakuya D., Itonga S., Mbaria J., Muthee J., Musau J. (2013). Ethnobotanical Survey of Biopesticides and Other Medicinal Plants Traditionally Used in Meru Central District of Kenya. J. Ethnopharmacol..

[64] Ali A.D., Ior L.D., Dogo G.A., Joshua J.I., Gushit J.S. (2022). Ethnobotanical Survey of Plants Used as Biopesticides by Indigenous People of Plateau State, Nigeria. Diversity.

[65] Kumar J., Ramlal A., Mallick D., Mishra V. (2021). An Overview of Some Biopesticides and Their Importance in Plant Protection for Commercial Acceptance. Plants.

[66] Fenibo E.O., Matambo T. (2025). Biopesticides for Sustainable Agriculture: Feasible Options for Adopting Cost-Effective Strategies. Front. Sustain. Food Syst..

[67] do Nascimento Junior D.R., Tabernero A., Cabral Albuquerque E.C.d.M., Vieira de Melo S.A.B. (2021). Biopesticide Encapsulation Using Supercritical CO_2_: A Comprehensive Review and Potential Applications. Molecules.

[68] Agboola A.R., Okonkwo C.O., Agwupuye E.I., Mbeh G. (2022). Biopesticides and Conventional Pesticides: Comparative Review of Mechanism of Action and Future Perspectives. AROC Agric..

[69] Petrović S., Leskovac A. (2026). Biopesticides and Human Health Risks: A Critical Review. Toxics.

[70] Gautam S., Khanal S., Khanal D., Mishra S.R., Ghimire S. (2022). Phytochemical Screening of Selected Botanicals and Their Effectiveness Against Maize Weevil (*Sitophilus zeamais* Motsch.) at Paklihawa, Rupandehi, Nepal. Adv. J. Grad. Res..

[71] Abdallah E.M., Alhatlani B.Y., de Paula Menezes R., Martins C.H.G. (2023). Back to Nature: Medicinal Plants as Promising Sources for Antibacterial Drugs in the Post-Antibiotic Era. Plants.

[72] Said-Al Ahl H., Hikal W.M., Tkachenko K.G. (2017). Essential Oils with Potential as Insecticidal Agents: A Review. Int. J. Env. Plan. Manag..

[73] Ukoroije R.B., Otayor R.A. (2020). Review on the Bio-Insecticidal Properties of Some Plant Secondary Metabolites: Types, Formulations, Modes of Action, Advantages and Limitations. Asian J. Res. Zool..

[74] El-Saadony M.T., Saad A.M., Mohammed D.M., Alkafaas S.S., Abd El-Mageed T.A., Fahmy M.A., Ezzat Ahmed A., Algopishi U.B., Abu-Elsaoud A.M., Mosa W.F. (2025). Plant Bioactive Compounds: Extraction, Biological Activities, Immunological, Nutritional Aspects, Food Application, and Human Health Benefits—A Comprehensive Review. Front. Nutr..

[75] Hikal W.M., Baeshen R.S., Said-Al Ahl H.A. (2017). Botanical Insecticide as Simple Extractives for Pest Control. Cogent Biol..

[76] Chafiki S., Oukarroum A., Alouani M., Lahchimi H., Qessaoui R., El Assri S., El Arroussi H., Bouharroud R. (2026). Microbial-and Plant-Based Biopesticides for Management of Crop Pests and Diseases: A Bibliographical Analysis. J. Umm Al-Qura Univ. Appl. Sci..

[77] Lahlali R., Kouighat M., Khadiri M., Boutagayout A., Özer G., Laasli S.-E., Farhaoui A. (2025). Biopesticides for a Sustainable Agriculture: Prospects and Challenges in Disease Management. Physiol. Mol. Plant Pathol..

[78] Siraj J. (2022). Ethnobotany.

[79] Ishtiaq M., Sardar T., Hussain I., Maqbool M., Mazhar M.W., Parveen A., Ajaib M., Bhatti K.H., Hussain T., Gul A. (2024). Traditional Ethnobotanical Knowledge of Important Local Plants in Sudhnoti, Azad Kashmir, Pakistan. Sci. Rep..

[80] Nko K.I., Mpolokeng T.G., Mokgau K., Asong J.A., Omotayo A.O., Aremu A.O. (2024). Ethnobotanical, Biological, and Phytochemical Qualities of Locally Sourced Leafy Vegetables for Food Security, Good Health and General Well-Being in South Africa: A Review. S. Afr. J. Bot..

[81] Suleiman M., Rugumamu C.P., Ibrahim N.D. (2018). Repellency Potential of Some Botanicals against the Maize Weevil, *Sitophilus zeamais* (Motschulsky, 1855) (Coleoptera: Curculionidae) in Stored Sorghum. Pol. J. Entomol..

[82] Arannilewa S., Ekrakene T., Akinneye J. (2006). Laboratory Evaluation of Four Medicinal Plants as Protectants against the Maize Weevil, *Sitophilus zeamais* (Mots). Afr. J. Biotechnol..

[83] Ndungu M., Lwande W., Hassanali A., Moreka L., Chhabra S.C. (1995). Cleome Monophylla Essential Oil and Its Constituents as Tick (*Rhipicephalus appendiculatus*) and Maize Weevil (*Sitophilus zeamais*) Repellents. Entomol. Exp. Appl..

[84] Hiruy B., Getu E. (2018). Efficacy of Solvent Extracts of *Calpurnia aurea* (Ait.) Benth and *Milletia ferruginea* (Hochest) Baker Leaves against Maize Weevils, *Sitophilus zeamais* (Motsch.) of Stored Maize in Ethiopia. J. Stored Prod. Postharvest Res..

[85] Hiruy B., Getu E. (2023). Evaluation of Calpurnia Aurea Leaf Extracts as Natural Insect Repellents for Stored Product Insect Pests in Ethiopia. Life.

[86] Ukeh D.A., Birkett M.A., Pickett J.A., Bowman A.S., Mordue A.J. (2009). Repellent Activity of Alligator Pepper, *Aframomum melegueta*, and Ginger, Zingiber Officinale, against the Maize Weevil, *Sitophilus zeamais*. Phytochemistry.

[87] Liu Z.L., Chu S.S., Liu Q.R. (2010). Chemical Composition and Insecticidal Activity against Sitophilus Zeamais of the Essential Oils of *Artemisia capillaris* and *Artemisia mongolica*. Molecules.

[88] Akinneye J.O., Ogungbite O.C. (2013). Insecticidal Activities of Some Medicinal Plants against *Sitophilus zeamais* (Motschulsky) (Coleoptera: Curculionidae) on Stored Maize. Arch. Phytopathol. Plant Prot..

[89] Mohammad M.Y., Haniffa H.M., Sujarajiini V. (2023). Insecticidal Effect of Selected Medicinal Plants on *Sitophilus zeamais* Mostschulsky in Stored Maize. Biocatal. Agric. Biotechnol..

[90] David L., Olajide O.O., Toluwalase A.D. (2025). Laboratory Evaluation of Spice Powders and Extracts for Biocontrol of Maize Weevil (*Sitophilus zeamais*) in Stored Maize Seeds. Res. J. Bot..

[91] Majdoub S., Chaabane-Banaoues R., Mokni R.E., Chaieb I., Piras A., Porcedda S., Hammami S. (2022). Composition, Insecticidal and Antifungal Activities of Tunisian *Daucus setifolius* Essential Oil. Waste Biomass Valoris..

[92] Seepe H.A., Lodama K.E., Sutherland R., Nxumalo W., Amoo S.O. (2020). In Vivo Antifungal Activity of South African Medicinal Plant Extracts against Fusarium Pathogens and Their Phytotoxicity Evaluation. Plants.

[93] Sanchez Matías M.d.H., Gómez A.d.l.A., Jiménez C.M., Tanguy Guillo S., Aristimuño Ficoseco M.E.d.M., Catalán C.A., Grougnet R., Kritsanida M., Sampietro D.A. (2025). Antifungal Activity of Extracts from Justicia Species against *Fusarium graminearum*. Nat. Prod. Res..

[94] Rashad Y.M., Aseel D.G., Hafez E.E. (2018). Antifungal Potential and Defense Gene Induction in Maize against Rhizoctonia Root Rot by Seed Extract of *Ammi visnaga* (L.) Lam. Phytopathol. Mediterr..

[95] Magro A., Carolino M., Bastos M., Mexia A. (2006). Efficacy of Plant Extracts against Stored Products Fungi. Rev. Iberoam. Micol..

[96] Onyewuchi O., Ihejirika G., Alagba A., Chinyere I. (2016). Effects of some plant extracts on the *Curvularia* leaf spot of maize. Int. J. Agric. Rural. Dev..

[97] Channya F.K., Chimbekujwo I., Talba U., Zakari B.G. (2023). Control of Ear Rot Fungal Disease of Maize (*Zea mays* L.) Caused by *Fusarium graminearum* Using Plant Extracts. AROC Nat. Prod. Res..

[98] Mungwari C.P., King’ondu C.K., Sigauke P., Obadele B.A. (2025). Conventional and Modern Techniques for Bioactive Compounds Recovery from Plants. Sci. Afr..

[99] Park K. (2023). The Role of Dietary Phytochemicals: Evidence from Epidemiological Studies. Nutrients.

[100] Hossain M.S., Wazed M.A., Asha S., Amin M.R., Shimul I.M. (2025). Dietary Phytochemicals in Health and Disease: Mechanisms, Clinical Evidence, and Applications—A Comprehensive Review. Food Sci. Nutr..

[101] Rodríguez-Negrete E.V., Morales-González Á., Madrigal-Santillán E.O., Sánchez-Reyes K., Álvarez-González I., Madrigal-Bujaidar E., Valadez-Vega C., Chamorro-Cevallos G., García-Melo L.F., Morales-González J.A. (2024). Phytochemicals and Their Usefulness in the Maintenance of Health. Plants.

[102] Kumar A., P N., Kumar M., Jose A., Tomer V., Oz E., Proestos C., Zeng M., Elobeid T., K S. (2023). Major Phytochemicals: Recent Advances in Health Benefits and Extraction Method. Molecules.

[103] Dincheva I., Badjakov I., Galunska B. (2023). New Insights into the Research of Bioactive Compounds from Plant Origins with Nutraceutical and Pharmaceutical Potential. Plants.

[104] Greff B., Sáhó A., Lakatos E., Varga L. (2023). Biocontrol Activity of Aromatic and Medicinal Plants and Their Bioactive Components against Soil-Borne Pathogens. Plants.

[105] Gindaba A., Negeri M., Abdisa B., Nemo R., Kitila C. (2024). Phytochemical Screening and Insecticidal Activities of Some Medicinal Plants against the Maize Weevil, *Sitophilus zeamais* (Motschulsky) (Coleoptera: Curculionidae). Sci. Rep..

[106] Rodríguez A., Beato M., Usseglio V.L., Camina J., Zygadlo J.A., Dambolena J.S., Zunino M.P. (2022). Phenolic compounds as controllers of Sitophilus zeamais: A look at the structure-activity relationship. J. Stored Prod. Res..

[107] Acheuk F., Basiouni S., Shehata A.A., Dick K., Hajri H., Lasram S., Yilmaz M., Emekci M., Tsiamis G., Spona-Friedl M. (2022). Status and Prospects of Botanical Biopesticides in Europe and Mediterranean Countries. Biomolecules.

[108] Lin D., Xiao M., Zhao J., Li Z., Xing B., Li X., Kong M., Li L., Zhang Q., Liu Y. (2016). An Overview of Plant Phenolic Compounds and Their Importance in Human Nutrition and Management of Type 2 Diabetes. Molecules.

[109] Ninkuu V., Aluko O.O., Yan J., Zeng H., Liu G., Zhao J., Li H., Chen S., Dakora F.D. (2025). Phenylpropanoids Metabolism: Recent Insight into Stress Tolerance and Plant Development Cues. Front. Plant Sci..

[110] Rabizadeh F., Mirian M.S., Doosti R., Kiani-Anbouhi R., Eftekhari E. (2022). Phytochemical Classification of Medicinal Plants Used in the Treatment of Kidney Disease Based on Traditional Persian Medicine. Evid.-Based Complement. Altern. Med..

[111] Lahlali R., El Hamss H., Mediouni-Ben Jemâa J., Barka E.A. (2022). The Use of Plant Extracts and Essential Oils as Biopesticides. Front. Agron..

[112] Ayllón-Gutiérrez R., Díaz-Rubio L., Montaño-Soto M., Haro-Vázquez M.d.P., Córdova-Guerrero I. (2024). Applications of Plant Essential Oils in Pest Control and Their Encapsulation for Controlled Release: A Review. Agriculture.

[113] Yallappa R., Eraiah K.H., Babu C.S.V. (2025). Chemical and Biological Investigation of *Plectranthus amboinicus* Essential Oil in the Control of *Sitophilus oryzae* L. Sci. Rep..

[114] Chaudhary S., Yadav S.K., Verma P. (2024). Botanical Insecticides for Crop Protection: Major Classes and Possible Mechanisms of Action. Insecticides in Pest Control-Impact, Challenges and Strategies: Impact, Challenges and Strategies.

[115] Bakkali F., Averbeck S., Averbeck D., Idaomar M. (2008). Biological Effects of Essential Oils—A Review. Food Chem. Toxicol..

[116] Soujanya P.L., Sekhar J., Kumar P., Sunil N., Prasad C.V., Mallavadhani U. (2016). Potentiality of Botanical Agents for the Management of Post-Harvest Insects of Maize: A Review. J. Food Sci. Technol..

[117] Aremu A.O., Omogbene T.O., Fadiji T., Lawal I.O., Opara U.L., Fawole O.A. (2024). Plants as an Alternative to the Use of Chemicals for Crop Protection against Biotic Threats: Trends and Future Perspectives. Eur. J. Plant Pathol..

[118] Assadpour E., Can Karaça A., Fasamanesh M., Mahdavi S.A., Shariat-Alavi M., Feng J., Kharazmi M.S., Rehman A., Jafari S.M. (2024). Application of Essential Oils as Natural Biopesticides; Recent Advances. Crit. Rev. Food Sci. Nutr..

[119] Devi K.C., Devi S.S. (2013). Insecticidal and Oviposition Deterrent Properties of Some Spices against Coleopteran Beetle, Sitophilus Oryzae. J. Food Sci. Technol..

[120] Ogendo J., Kostyukovsky M., Ravid U., Matasyoh J., Deng A., Omolo E., Kariuki S., Shaaya E. (2008). Bioactivity of *Ocimum gratissimum* L. Oil and Two of Its Constituents against Five Insect Pests Attacking Stored Food Products. J. Stored Prod. Res..

[121] Timilsena Y.P., Phosanam A., Stockmann R. (2023). Perspectives on Saponins: Food Functionality and Applications. Int. J. Mol. Sci..

[122] Riaz M., Khalid R., Afzal M., Anjum F., Fatima H., Zia S., Rasool G., Egbuna C., Mtewa A.G., Uche C.Z. (2023). Phytobioactive Compounds as Therapeutic Agents for Human Diseases: A Review. Food Sci. Nutr..

[123] Tlak Gajger I., Dar S.A. (2021). Plant Allelochemicals as Sources of Insecticides. Insects.

[124] Salim R., Nehvi I., Mir R., Tyagi A., Ali S., Bhat O. (2023). A Review on Anti-Nutritional Factors: Unraveling the Natural Gateways to Human Health. Front. Nutr..

[125] Guo C., Wang L., Chen N., Zhang M., Jia J., Lv L., Li M. (2024). Advances in Research and Utilisation of Botanical Pesticides for Agricultural Pest Management in Inner Mongolia, China. Chin. Herb. Med..

[126] Ntalli N.G., Menkissoglu-Spiroudi U. (2011). Pesticides of Botanical Origin: A Promising Tool in Plant Protection. Pesticides: Formulations, Effects, Fate.

[127] Pawase P.A., Goswami C., Shams R., Pandey V.K., Tripathi A., Rustagi S. (2024). A Conceptual Review on Classification, Extraction, Bioactive Potential and Role of Phytochemicals in Human Health. Future Foods.

[128] Varela C., Silva F., Costa G., Cabral C. (2023). Alkaloids: Their Relevance in Cancer Treatment. New Insights into Glioblastoma.

[129] Şengül Demirak M.Ş., Canpolat E. (2022). Plant-Based Bioinsecticides for Mosquito Control: Impact on Insecticide Resistance and Disease Transmission. Insects.

[130] Muñoz I.J., Schilman P.E., Barrozo R.B. (2020). Impact of Alkaloids in Food Consumption, Metabolism and Survival in a Blood-Sucking Insect. Sci. Rep..

[131] Qiao K., Zhao M., Huang Y., Liang L., Zhang Y. (2024). Bitter Perception and Effects of Foods Rich in Bitter Compounds on Human Health: A Comprehensive Review. Foods.

[132] Pizzi A. (2021). Tannins Medical/Pharmacological and Related Applications: A Critical Review. Sustain. Chem. Pharm..

[133] Govindarajan R., Revathi S., Rameshkumar N., Krishnan M., Kayalvizhi N. (2016). Microbial Tannase: Current Perspectives and Biotechnological Advances. Biocatal. Agric. Biotechnol..

[134] Siddiqui A., Moid H. (2022). An Introduction on Phytochemical Analysis and Their Types. Sch. Res. Libr..

[135] Mora J., Pott D.M., Osorio S., Vallarino J.G. (2022). Regulation of Plant Tannin Synthesis in Crop Species. Front. Genet..

[136] Zayed A., Abdelkareem S., Talaat N., Dayem D.A., Farag M.A. (2025). Tannin in Foods: Classification, Dietary Sources, and Processing Strategies to Minimize Anti-Nutrient Effects. Food Bioprocess. Technol..

[137] Ullah A., Munir S., Badshah S.L., Khan N., Ghani L., Poulson B.G., Emwas A.-H., Jaremko M. (2020). Important Flavonoids and Their Role as a Therapeutic Agent. Molecules.

[138] Janabi A.H.W., Kamboh A.A., Saeed M., Xiaoyu L., BiBi J., Majeed F., Naveed M., Mughal M.J., Korejo N.A., Kamboh R. (2020). Flavonoid-Rich Foods (FRF): A Promising Nutraceutical Approach against Lifespan-Shortening Diseases. Iran. J. Basic. Med. Sci..

[139] Mutha R.E., Tatiya A.U., Surana S.J. (2021). Flavonoids as Natural Phenolic Compounds and Their Role in Therapeutics: An Overview. Future J. Pharm. Sci..

[140] Liga S., Paul C., Péter F. (2023). Flavonoids: Overview of Biosynthesis, Biological Activity, and Current Extraction Techniques. Plants.

[141] Chen S., Wang X., Cheng Y., Gao H., Chen X. (2023). A Review of Classification, Biosynthesis, Biological Activities and Potential Applications of Flavonoids. Molecules.

[142] Dias M.C., Pinto D.C., Silva A.M. (2021). Plant Flavonoids: Chemical Characteristics and Biological Activity. Molecules.

[143] Verghese A., Soumya C., Shivashankar S., Manivannan S., Krishnamurthy S. (2012). Phenolics as Chemical Barriers to Female Fruit Fly, Bactrocera Dorsalis (Hendel) in Mango. Curr. Sci..

[144] War A.R., Paulraj M.G., Ahmad T., Buhroo A.A., Hussain B., Ignacimuthu S., Sharma H.C. (2012). Mechanisms of Plant Defense against Insect Herbivores. Plant Signal. Behav..

[145] Pratyusha S. (2022). Phenolic Compounds in the Plant Development and Defense: An Overview. Plant Stress Physiology—Perspectives in Agriculture.

[146] Khanam S., Mishra P., Faruqui T., Alam P., Albalawi T., Siddiqui F., Rafi Z., Khan S. (2025). Plant-Based Secondary Metabolites as Natural Remedies: A Comprehensive Review on Terpenes and Their Therapeutic Applications. Front. Pharmacol..

[147] Masyita A., Sari R.M., Astuti A.D., Yasir B., Rumata N.R., Emran T.B., Nainu F., Simal-Gandara J. (2022). Terpenes and Terpenoids as Main Bioactive Compounds of Essential Oils, Their Roles in Human Health and Potential Application as Natural Food Preservatives. Food Chem. X.

[148] de Oliveira J.L., Campos E.V.R., Bakshi M., Abhilash P., Fraceto L.F. (2014). Application of Nanotechnology for the Encapsulation of Botanical Insecticides for Sustainable Agriculture: Prospects and Promises. Biotechnol. Adv..

[149] Luneja R.L., Mkindi A.G. (2025). Advances in Botanical-Based Nanoformulations for Sustainable Cotton Insect Pest Management in Developing Countries. Front. Agron..

[150] Kutasy B., Hegedűs G., Kiniczky M., Pallos J.P., Nagy Á., Pócsi I., Pákozdi K., Kállai M., Weingart C., Andor K. (2025). Garlic Extracts Nanoliposome as an Enhancer of Bioavailability of ABA and Thiamine Content and as an Antifungal Agent Against *Fusarium oxysporum* f. Sp. Pisi Infecting Pisum Sativum. Agronomy.

[151] Mukhtar T. (2025). Plant Immunity and Resistance Mechanisms: An Editorial. Physiol. Mol. Plant Pathol..

[152] Oide S., Bejai S., Staal J., Guan N., Kaliff M., Dixelius C. (2013). A Novel Role of PR 2 in Abscisic Acid (ABA) Mediated, Pathogen-induced Callose Deposition in Arabidopsis Thaliana. New Phytol..

[153] Erb M., Reymond P. (2019). Molecular Interactions between Plants and Insect Herbivores. Annu. Rev. Plant Biol..

[154] Yang Z., Zhi P., Chang C. (2022). Priming Seeds for the Future: Plant Immune Memory and Application in Crop Protection. Front. Plant Sci..

[155] Kasali F.M., Tusiimire J., Kadima J.N., Agaba A.G. (2021). Ethnomedical Uses, Chemical Constituents, and Evidence-Based Pharmacological Properties of *Chenopodium ambrosioides* L.: Extensive Overview. Future J. Pharm. Sci..

[156] Cortese D., Mareco Da Silva M.M., de Oliveira G.S., Mussury R.M., Fernandes M.G. (2022). Repellency and Reduction of Offspring Emergence Potential of Some Botanical Extracts against *Sitophilus zeamais* (Coleoptera: Curculionidae) in Stored Maize. Insects.

[157] Suleiman M., Sani I., Yusuf A., Abdullahi B. (2019). Entomocidal Activity of Some Plant Extracts against *Sitophilus zeamais* Motschulsky (Coleoptera: Curculionidae). Afr. J. Agric. Res..

[158] Fernandes E.T., Favero S. (2014). Óleo Essencial de *Schinus molle* L. Para o Controle de *Sitophilus zeamais* Most. 1855 (Coleoptera: Curculionidae) Em Milho. Rev. Bras. Agroecol..

[159] Seepe H.A., Nxumalo W., Amoo S.O. (2021). Natural Products from Medicinal Plants against Phytopathogenic Fusarium Species: Current Research Endeavours, Challenges and Prospects. Molecules.

[160] Gómez-Lama Cabanás C., Mercado-Blanco J. (2025). Groundbreaking Technologies and the Biocontrol of Fungal Vascular Plant Pathogens. J. Fungi.

[161] Pan X., Guo X., Zhai T., Zhang D., Rao W., Cao F., Guan X. (2023). Nanobiopesticides in Sustainable Agriculture: Developments, Challenges, and Perspectives. Environ. Sci. Nano.

[162] Ngangom L., Shabaaz Begum J., Gautam S., Venugopal D., Joshi S. (2024). Nanotechnology in Food Crop Production and Food Processing Industry. Food Production, Diversity, and Safety Under Climate Change.

[163] Kah M., Hofmann T. (2014). Nanopesticide Research: Current Trends and Future Priorities. Environ. Int..

[164] Ghosh A., Majumdar D., Biswas H., Chowdhury A., Podder S. (2025). Nano-Biopesticide Formulation Comprising of Silver Nanoparticles Anchored to Ocimum Sanctum: A Sustainable Approach to Pest Control in Jute Farming. Sci. Rep..

[165] Jali P., Acharya S., Mahalik G. (2024). Antimicrobial Efficacy of Nano-Particles for Crop Protection and Sustainable Agriculture. Discov. Nano.

[166] Vinci G., Savastano M., Restuccia D., Ruggeri M. (2025). Nanobiopesticides: Sustainability Aspects and Safety Concerns. Environments.

[167] Mawcha K.T., Malinga L., Muir D., Ge J., Ndolo D. (2025). Recent Advances in Biopesticide Research and Development with a Focus on Microbials. F1000Research.

[168] Jomantas T., Kemzūraitė A., Steponavičius D., Andriušis A., Dorelis M., Balčiūnas J. (2025). Management Measures for the Mitigation of Spray Drift of Very Fine Droplets Sprayed by a Spraying Robot. Sci. Rep..

[169] Assalin M.R., de Castro S.C., Mioti M.V., dos Santos V.T., Fazolin M., Forim M.R., do Nascimento Queiroz S.C., Marinho-Prado J.S., Tasic L. (2025). Nanoencapsulation of Essential Oil of *Piper aduncum*: Evaluation of Insecticidal Activity and Phytotoxicity of a Botanical Pesticide. Plant Nano Biol..

[170] Wang X., Xie H., Wang P., Yin H. (2023). Nanoparticles in Plants: Uptake, Transport and Physiological Activity in Leaf and Root. Materials.

[171] Islam S. (2025). Toxicity and Transport of Nanoparticles in Agriculture: Effects of Size, Coating, and Ageing. Front. Nanotechnol..

[172] Alwora G.O., Gichuru E.K., Ogendo J.O., Okumu O.O. (2026). An Overview of Biopesticides Use and Analysis of the Kenyan Legal Frameworks Regulating Biocontrol Agents: A Review. Front. Sustain. Food Syst..

[173] Patel M., Surti M., Janiyani K., Adnan M. (2025). Next-Generation Nanotechnology-Integrated Biosurfactants: Innovations in Biopesticide Development for Sustainable and Modern Agriculture. Adv. Colloid Interface Sci..

[174] Kutasy-Takács B., Pallos J.P., Kiniczky M., Hegedűs G., Virág E. (2026). Plant-Derived Biostimulants and Liposomal Formulations in Sustainable Crop Protection and Stress Tolerance. Appl. Sci..

[175] Chaud M., Souto E.B., Zielinska A., Severino P., Batain F., Oliveira-Junior J., Alves T. (2021). Nanopesticides in Agriculture: Benefits and Challenge in Agricultural Productivity, Toxicological Risks to Human Health and Environment. Toxics.

[176] Yousef H.A., Fahmy H.M., Arafa F.N., Abd Allah M.Y., Tawfik Y.M., El Halwany K.K., El-Ashmanty B.A., Al-Anany F.S., Mohamed M.A., Bassily M.E. (2023). Nanotechnology in Pest Management: Advantages, Applications, and Challenges. Int. J. Trop. Insect Sci..

[177] Prieto C., Lagaron J.M. (2024). Nanoencapsulation and Nanocoating of Bioactives of Application Interest in Food, Nutraceuticals and Pharma. Nanomaterials.

